# Ensemble-based design of tau to inhibit aggregation while preserving biological activity

**DOI:** 10.21203/rs.3.rs-3796916/v1

**Published:** 2024-01-16

**Authors:** Sofia Bali, Ruhar Singh, Pawel M. Wydorski, Aleksandra Wosztyl, Valerie A. Perez, Dailu Chen, Josep Rizo, Lukasz A. Joachimiak

**Affiliations:** 1 Molecular Biophysics Graduate Program, University of Texas Southwestern Medical Center, Dallas, TX 75390, United States; 2 Center for Alzheimer’s and Neurodegenerative Diseases, Peter O’Donnell Jr. Brain Institute, University of Texas; 3 Department of Biophysics, University of Texas Southwestern Medical Center, Dallas, TX 75390, United States Southwestern Medical Center, Dallas, TX 75390, United States; 4 Department of Biochemistry, University of Texas Southwestern Medical Center, Dallas, TX 75390, United States; 5 Department of Pharmacology, University of Texas Southwestern Medical Center, Dallas, TX 75390, United States

## Abstract

The microtubule-associated protein tau is implicated in neurodegenerative diseases characterized by amyloid formation. Mutations associated with frontotemporal dementia increase tau aggregation propensity and disrupt its endogenous microtubule-binding activity. The structural relationship between aggregation propensity and biological activity remains unclear. We employed a multi-disciplinary approach, including computational modeling, NMR, cross-linking mass spectrometry, and cell models to design tau sequences that stabilize its structural ensemble. Our findings reveal that substitutions near the conserved ‘PGGG’ beta-turn motif can modulate local conformation, more stably engaging in interactions with the ^306^VQIVYK^311^ amyloid motif to decrease aggregation in vitro and in cells. Designed tau sequences maintain microtubule binding and explain why 3R isoforms of tau exhibit reduced pathogenesis over 4R isoforms. We propose a simple mechanism to reduce the formation of pathogenic species while preserving biological function, offering insights for therapeutic strategies aimed at reducing protein misfolding in neurodegenerative diseases.

## INTRODUCTION

The deposition of the microtubule-associated protein tau is linked to many neurodegenerative diseases commonly referred to as tauopathies^1-3^. Recent cryo-EM tau fibril structures derived from patient material uncovered how tau adopts distinct conformations (i.e., structural polymorphs) across different tauopathies^4-6^, each able to propagate in a prion-like manner^7,8^. The mechanism for tau misfolding into different conformations in each disease is poorly understood. Despite this link to disease, tau is also remarkably aggregation-resistant, and the mechanisms that regulate its intrinsic propensity to remain soluble and the factors that influence its aggregation propensity remain unknown. Prior studies have shown that aggregation-resistant tau can convert into an aggregation-prone conformation, termed a seed, which may recruit inert tau monomers to form amyloid fibrils^9^. These studies suggest that these seeds can be isolated from Alzheimer’s disease patient material^10^. In tauopathy mouse models, these small seeds are detected in young mice prior to insoluble species and even soluble oligomers, underscoring the central role of seeds in disease progression^11^. Structural and biochemical studies comparing inert tau monomers and monomeric seeds identify rearrangements of long-range charge complementary interactions and short-range hydrophobic interactions^12^. The hydrophobic interactions are mediated by amyloidogenic motifs essential for tau fibril formation, including the ^275^VQIINK^280^, ^306^VQIVYK^311^, and ^337^VEVKSE^342^ amyloid motifs^13,14^. Interestingly, in all tau fibril structures, recombinant and disease-derived, the ^306^VQIVYK^311^ amyloid motif stabilizes the fibrillar assemblies^14,15^, while mutations in this motif abrogate tau aggregation^10^. Additionally, time-resolved cryo-EM analysis on disease-specific tau folds uncovered early intermediates stabilized by only VQIVYK-VQVIYK interactions^16^. This mechanism suggests that the combinatorics of amyloid motif exposure and subsequent stabilization of key interactions determine the formation of a given fibrillar fold. It remains unknown how this conversion towards specific polymorphs occurs in disease. Still, cofactor binding or post-translational modifications may be involved in this process^17-20^. Efforts to understand how to further impair tau aggregation by locally stabilizing amyloid motif interactions may help produce tau sequences that retain biological activity but encode additional structural features that resist conversion into seeds.

Tau encodes a region with four pseudo-repeats, referred to as the tau repeat domain (tauRD), which defines the minimal region required for binding to microtubules (MT)^21^. Structural studies of tauRD bound to MT revealed that it binds in a mostly extended conformation spanning tubulin interfaces, enabling MT stabilization^22-24^. Mutations in the *MAPT* gene that encodes tau are linked to frontotemporal dementia (FTD)^25,26^, and a majority localize to the repeat domain of tau^27,28^. Their effect has been proposed to be two-fold: a) decrease affinity for MTs, thus producing a larger pool of cytosolic tau, and b) modify the conformation of tau to promote aggregation. Alternative splicing of the *MAPT* gene results in two isoforms of the tau repeat domain that have differential aggregation propensity, 4R tauRD (243-380) and 3R tauRD (243-380, ΔR2:275-305)^29^. In early development, the second repeat (R2:275-305) is spliced out in humans, producing predominantly the 3R tau isoform, while later in life, 4R tau isoforms dominate^30^. The 4R tau isoforms have been shown to aggregate with a higher propensity compared to 3R tau isoforms^31^. Tau amyloid deposits in tauopathies are most commonly composed of either a mixture of 4R and 3R or 4R-only isoforms, with Pick’s disease being the only tauopathy exclusively linked to 3R tau isoform^1,32^. Consistent with this idea, the reduction of 4R tau isoforms reduces tau pathology and toxicity in mouse models; by contrast, the reduction of 3R isoforms increases tau pathology^31^. This differential behavior of 3R and 4R tau isoforms presents avenues for therapeutic intervention, such as strategies underway using anti-sense oligonucleotides^32,33^. Importantly, mechanistic knowledge of how these isoforms change intrinsic properties of tau to aggregate will suggest novel approaches to regulate the aggregation of 4R tau isoforms. A proline to serine or leucine mutation at position 301 causes FTD^28^ and is commonly used in animal, cellular, and in vitro models to study tau aggregation^34-36^. This mutation localizes to the proline in the ^301^PGGG^304^ motif that stabilizes beta-turns upstream of the ^306^VQIVYK^311^ amyloid motif^37,38^. Recent cryo-EM structures of P301S tau aggregates isolated from tauopathy mouse models show conformations in this region similar to PSP and GPT structures but different from CBD, AGD, and GGT^39^, revealing how essential and conformationally versatile this region is in tau aggregation. Our previous studies using minimal fragments from the 4R tau isoform established that the ^294^KDNIKHVPGGGS^305^ sequence upstream of ^306^VQIVYK^311^ is sufficient to regulate amyloid motif aggregation^13^. We observed that the sequence upstream of the ‘PGGG,’ ‘KDNIKHV’ can transiently engage with ^306^VQIVYK^311^ and is dependent on the ‘PGGG’ sequence forming a beta-turn, which the P301S mutation can disrupt^13,36,40^. However, the equivalent mutation, P270S, in 3R tauRD element ^263^TENLKHQPGGGK^274^ does not have this effect^13^. Additionally, we have recently shown that acetylation of lysines in proximity to amyloid motifs destabilizes the protective features, enabling faster tau self-association into fibrils^18^. Thus, a deeper investigation into how localized sequences change conformation and how they regulate ^306^VQIVYK^311^-based tau aggregation will yield new insight into how to tune tau ensembles to inhibit assembly into defined fibril conformations.

Here, we use a multidisciplinary approach to understand how local interactions with ^306^VQIVYK^311^ regulate the aggregation propensity of tau. We first demonstrate enhancement in aggregation driven by a P301S mutation in the conserved ‘PGGG’ motif in 4R but not 3R tau fragments. Using solution NMR, we measure secondary structure propensities to show beta-turn signatures for the aggregation-resistant wild-type and mutant 3R and wild-type 4R fragments while the aggregation-prone mutant 4R fragment adopts a more extended conformation. We then employ a molecular dynamics (MD) framework and Markov state models (MSMs) to capture rapid local rearrangements that transition into beta-hairpin-like structures independent of the starting conformation in the aggregation-resistant sequences. In contrast, the mutant 4R fragment transitions to more diverse local structures, reaching unique unfolded states consistent with the NMR experiments. Next, we translate our results to tauRD and show that only a subset of the changes are required to maintain the aggregation suppression phenotype and, importantly, do not alter the binding kinetics to MTs consistent with modeling of the MT:tau complex. Finally, we validate our observations in cells to show that minimal mutations in the 4R tau isoform can readily slow down tau aggregation compared to WT 4R tau. Our data collectively supports that local interactions involving the VQIVYK amyloid motif play an important role in regulating its aggregation and provide a structural rationale for further inhibiting tau aggregation while maintaining biological function.

## RESULTS

### Structural propensities of the tau amyloid motif ^306^VQIVYK^311^ differ across 3R and 4R tau isoforms.

**Figure 1.**
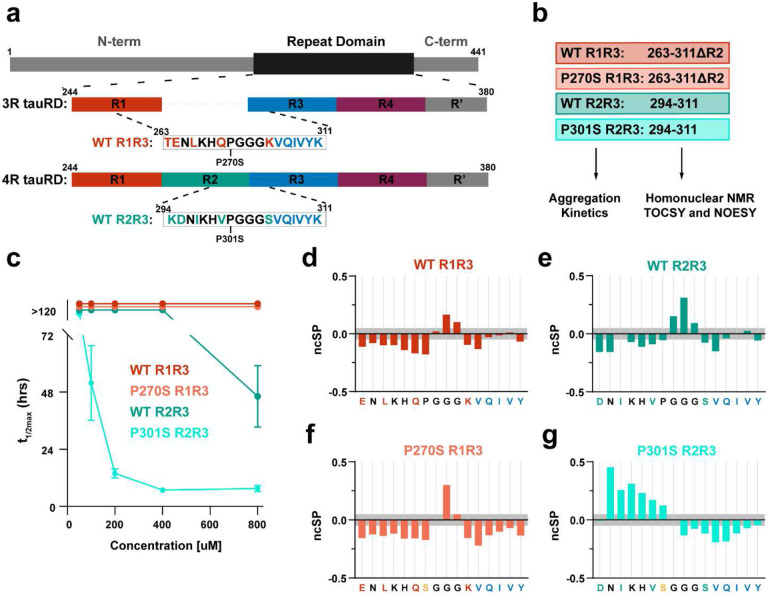
Proline to serine mutations in a 4R-like fragment leads to more unfolded ensembles. **a.** Cartoon schematic of 3R and 4R-like tau fragments. Repeat domains are colored red, green, blue, and purple of repeats 1, 2, 3, and 4. Inset shows WT minimal fragments that span repeats 1 and 3 and repeats 2 and 3. Site of the proline to serine mutation is indicated below the sequence. **b.** Schematic illustrating the experiments to probe aggregation and secondary structure of the four tested sequences: WT R1R3_263-311ΔR2_ (red), P270S R1R3_263-311ΔR2_ (orange), WT R2R3_295-311_ (green), and P301S R2R3_295-311_ (cyan). **c.** ThT fluorescence aggregation assay of WT R1R3_263-311ΔR2_ (red), P270S R1R3_263-311ΔR2_ (orange), WT R2R3_295-311_ (green), and P301S R2R3_295-311_ (cyan) peptides carried out at 5 concentrations: 50, 100, 200, 400, and 800 μM. Data is shown as the t_1/2max_ fit to each aggregation curve. The data is plotted as an average with a standard deviation. The curves are colored by peptide as in (b). **d-g**. neighbor corrected structure propensity (ncSP) calculated from peptide HA chemical shift assignments using ncSPC calculator for WT R1R3_263-311ΔR2_ (d, red), P270S R1R3_263-311ΔR2_ (e, green), WT R2R3_295-311_ (f, orange), and P301S R2R3_295-311_ (f, cyan). Negative/positive values correspond to β-sheet/α-helical propensity. Gray bars highlight the deviations expected for random coil structures.

The 4R and 3R tau isoforms differ in sequence upstream of the ^306^VQIVYK^311^ motif at only five out of 11 amino acids (Fig. 1a). To understand how sequence changes alter conformational ensembles to tune VQIVYK-based aggregation, we employed a combination of in vitro aggregation experiments, and homonuclear NMR spectroscopy (Fig. 1b). We first compared aggregation propensities of wild-type and FTD-linked proline to serine mutants in 4R and 3R peptides that span the inter-repeat regions between repeats 2 and 3 (WT R2R3_294-311_; **KD**N**I**KH**V**PGGG**S**VQIVYK and P301S R2R3_294-311_; **KD**N**I**KH**V**SGGG**S**VQIVYK) and repeats 1 and 3 (WT R1R3_263-311ΔR2_; **TE**N**L**KH**Q**PGGG**K**VQIVYK and P270S R1R3_263-311ΔR2_; **TE**N**L**KH**Q**SGGG**K**VQIVYK). We evaluated the aggregation propensity of the peptides in a Thioflavin T (ThT) fluorescence aggregation assay in a concentration series and fit the resultant curves to estimate a t_1/2max_ (Fig. 1c and Supplementary Fig. 1a-d). We determine that the P301S R2R3_295-311_ peptide assembled into amyloid fibrils rapidly and even at low concentrations starting at 100 μM (100 μM t_1/2max_ =52±14 h, 800 μM t_1/2max_ =7.5±1 h); peptide WT R2R3_295-311_ only aggregated at the highest concentration (800 μM t_1/2max_ =46hrs±13 h); while peptides WT R1R3_295-311ΔR2_ and P270S R1R3_263-311ΔR2_ did not aggregate at any concentration within the time frame of the experiment (Fig. 1c and Supplementary Fig. 1a-d). Importantly, these peptides recapitulate aggregation phenotypes of 3R and 4R tauRD with the P270S and P301S mutations, respectively (Supplementary Fig. 1e).

To test if the structures sampled by the peptide recapitulate the local structure sampled by this fragment within the tauRD, we acquired TOCSY and NOESY spectra for the four peptides and assigned the proton chemical shifts to the sequence (Supplementary Fig. 1f-i). For the aggregation-prone peptide, P301S R2R3_295-311_, we confirmed that signal intensities did not change throughout the experiment (Supplementary Fig. 1j). The small chemical shift deviations with respect to random coil observed for all the peptides values suggest that disordered structures predominate with an only a small proportion of the population sampling more defined secondary structures (Supplementary Table 1). To estimate the extent of beta-sheet or alpha helical content in our fragments we used the neighbor-corrected secondary structure propensity calculator^41,42^ based on Cα proton chemical shifts (HA). The ncSPC uses the SSP algorithm, which represents backbone deviations from random coil relative to the expected deviations from a fully formed beta-sheet or alpha helix, with the addition of a neighbor correction that incorporates the average deviation expected for a fragment of that size and produces a neighbor corrected structural propensity (ncSP).^41,42^ We observe that the three non-aggregating peptides, WT R1R3_263-311ΔR2_, WT R2R3_295-311_, and P270S R1R3_263-311ΔR2_, exhibit similar secondary structure propensities (Fig. 1d-f). The first N-terminal six residues of WT R1R3_263-311ΔR2_ and P270S R1R3_263-311ΔR2_ (263-269; **TE**N**L**KH**Q**) and WT R2R3_295-311_ (295-300; **D**N**I**KH**V**) show negative ncSP consistent with an enhanced population of extended conformations compared to random coil (Fig. 1d-f). The central ‘PGGG’ motif (270-273/301-304) in both WT R1R3_263-311ΔR2_ and WT R2R3_295-311_ and the mutant ‘SGGG’ motif (270-273) in P270S R1R3_263-311ΔR2_ show positive ncSP deviations consistent with the glycine residues adopting conformations compatible with an α-helix and hence suggestive of a partially populated turn structure (Fig. 1d-f). The C-terminal region of the sequence corresponding to the VQIVYK amyloid motif (306-311) in WT R1R3_263-311ΔR2_ and WT R2R3_295-311_ shows ncSP near 0, indicating dominant sampling of random coil structures (Fig. 1d, e). Yet, this region exhibits negative values for the P270S R1R3_263-311ΔR2_ indicative of enhanced extended structures (Fig. 1f). Analysis of the P301S R2R3_295-311_ peptide revealed differences. Like the P270S R1R3_263-311ΔR2_ peptide, the C-terminal sequence of P301S R2R3_295-311_ also shows an enhanced population of extended structures (Fig. 1g). However, the N-terminal sequence had positive ncSP consistent with a distinct helical propensity (Fig. 1g). We next compared the secondary structure behavior of these fragments to the equivalent regions from 4R tauRD (bmrb 19253)^23^ and tau 225-324 (bmrb 50129)^43^. Comparable secondary structure propensities are observed in this fragment from both datasets, except for a higher population of extended structure for the C-terminal amyloid motif (Supplementary Fig. 1k, l). Similarly, we compared the P301S mutant peptides to available NMR chemical shifts for tau 225-324 encoding a proline to leucine mutation at position 301 (bmrb 50130)^43^. We find that the loss of the proline in the longer tau fragment results in positive deviations from random coil for a few residues of the N-terminal sequence (Supplementary Fig. 1m), which recapitulates to some extent the positive ncSP observed for the N-terminus of the P301S R2R3_295-311_ fragment (Fig. 1g). Additionally, in P301S R2R3_295-311_ there is a large negative deviation consistent with extended β-sheet signal in the central ‘LGGG’ region that extends into the C-terminal amyloid motif.

### In silico ensembles reveal differences that correlate with aggregation propensity.

**Figure 2.**
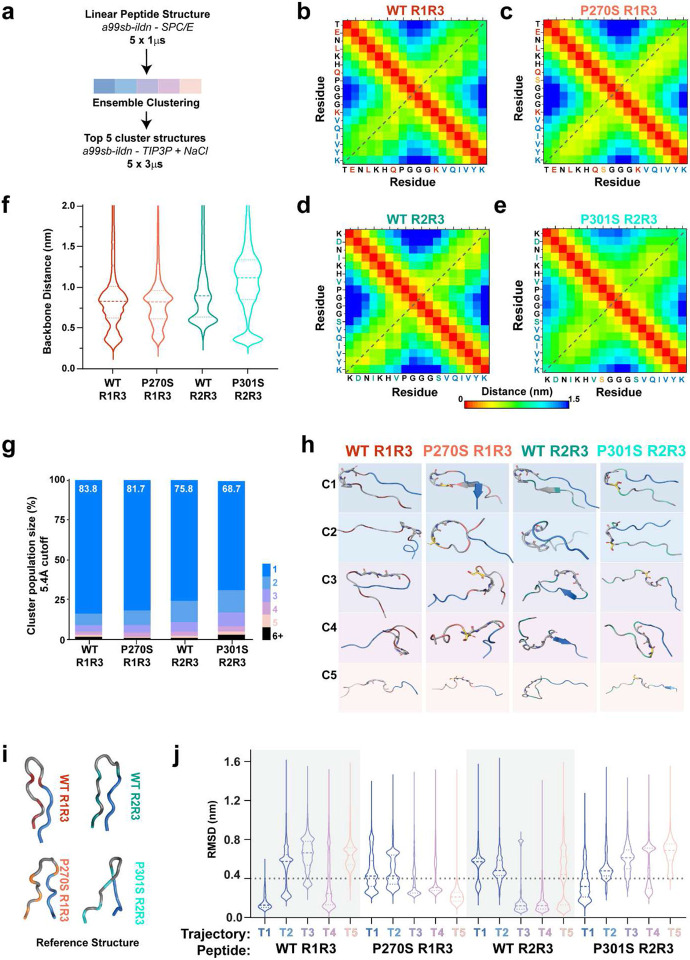
Building a structural ensemble of tau fragments reveals distinct behavior of aggregation prone 4R-derived peptide. **a.** Two stage MD simulation workflow for: WT R1R3_263-311ΔR2_, P270S R1R3_263-311ΔR2_, WT R2R3_295-311_, and P301S R2R3_295-311_. **b-e.** Average mean distance maps calculated from first step MD ensembles containing cumulative trajectories of the 5 replicates for WT R1R3_263-311ΔR2_ (h), P270S R1R3_263-311ΔR2_ (i), WT R2R3_295-311_ (j) and P301S R2R3_295-311_ (k). The maps are colored by distance from 0 nm in red to 1.5 nm in blue. **f.** Comparison of first step MD cumulative ensembles for WT R1R3_263-311ΔR2_ (red), P270S R1R3_263-311ΔR2_ (green), WT R2R3_295-311_ (orange), and P301S R2R3_295-311_ (cyan) by backbone distance between the center of mass of the N-term residues 1-6 (TENLKH/KDNIKH) and C-term amyloid motif residues (VQIVYK). The data is shown as violin plots with median and 25/75% quartiles labeled in dashes lines. **g.** Bar plots illustrating the cluster size for first step MD ensembles according to a 0.54nm cutoff. The clusters for each ensemble are colored blue to pink for clusters 1-5 with the remaining clusters colored black. **h.** Initial structures for second step MD simulations with 3 μs trajectories, from center structure of top 5 clusters (background color). Structures shown in cartoon representation with ‘VQIVYK’ colored in blue, unique residues colored as in Fig. 1b, and similar residues colored in gray. **i.** Second step MD ensembles top cluster structure according to a 0.4 nm cutoff. Shown as cartoon and colored as in h. **j.** Frequency distributions of RMSD to reference structure (shown in c) per trajectory (T1-T5) colored as in h. Clustering cutoff of 0.4 nm labeled as dashed line. Data shown as violin plots with median and 25/75% quartiles labeled in dashed lines.

The R1R3_263-311ΔR2_ and R2R3_295-311_ peptides only differ in 5 residues while exhibiting a distinct secondary structure behavior manifesting in different aggregation kinetics. We employed Molecular Dynamics (MD) simulations of these peptide model systems to assess intramolecular interactions. The four peptides (WT R2R3_295-311_, P301S R2R3_295-311_, WT R1R3_263-311ΔR2_, and P270S R1R3_263-311ΔR2_) were built as extended peptide chains that were minimized in GROMACS in only water (SPC/E) using the AMBER99sB-ILDN forcefield^44^. Minimized systems were run in 5 independent 1 μs simulations per peptide (Fig. 2a). The independent trajectories were concatenated, and from the resultant ensemble, we calculated the minimum residue pairwise distance for each of the four peptides (Fig. 2b-e). These distance maps reveal the interactions across the N-to-C terminal diagonal (Fig. 2b-e, gray dashed line) indicative of the compaction of the linear peptides. The WT R2R3_295-311_ and WT R1R3_263-311ΔR2_ peptides show symmetric interactions, centered at the dashed line with alternate patterning suggestive of a beta-hairpin fold centered at the PGGG motif (Fig. 1b, d). By contrast, in the serine mutant peptides, P270S R1R3_295-311ΔR2_ and P301S R2R3_295-311_ shorter distances are shifted below the diagonal and do not exhibit the alternate patterning (defined register) seen in the WT peptides (Fig. 2c, e). Intriguingly, in the aggregation-prone P301S R2R3_295-311_ peptide, we see larger distances between more residue pairs, consistent with a less defined fold (Fig. 1e).

We quantified the distance between the backbones of the C-terminal amyloid motif (‘VQIVYK’) and the N-terminal residues (‘TENLKH’ and ‘KDNIKH’) every 0.5ns, displaying the distances as population densities (Fig. 2f). Both WT R1R3_263-311ΔR2_ and P270S R1R3_263-311ΔR2_ had median backbone distances of approximately 0.8 nm, with a population of distances near the expected distance across two beta strands of 0.5 nm. In contrast, WT R2R3_295-311_ had a larger median distance of 0.9 nm derived from a bimodal distribution of states with distances around 0.6 nm and above 0.8 nm (Fig. 2f). P301S R2R3_295-311_ had the largest differences with a median backbone distance of 1.1 nm composed of a small proportion of conformations with backbone distances of around 0.4 nm, and the remaining population was expanded structures with distances >1.0 nm (Fig. 2f). These observations suggest that the aggregation-prone P301S R2R3_295-311_ samples more unfolded conformations. We clustered the ensembles to obtain representative structures using a cutoff distance of 0.54 nm (Fig. 2g). The largest cluster adopted a beta-hairpin-like conformation for all peptides (Fig. 2h, C1 structures), yet P301S R2R3_295-311_ had the smallest top cluster, 68.7% (Fig. 2g). Considering the aggregation behavior of the peptides reveals an ensemble distinction between the aggregation-resistant and aggregation-prone peptides.

Tau aggregation is not a spontaneous event in cells and requires additional perturbations such as interactions with an inducer, disease-associated mutations, or post-translational modifications. Thus, this aggregation resistance may suggest that the state on-pathway to forming amyloid is conformationally distant and may be more accessible in 4R-tau in the context of the P301S mutation. To probe these more distant states and increase our sampling of states corresponding to the solution NMR ensembles, we implemented longer simulations initiated from the mean structure of the top 5 clusters for the four peptides (Fig. 2h). Systems were minimized in water (TIP3) with the addition of Na^+^ and Cl^−^ counter ions at an estimated 150 mM ionic strength. Minimized systems were simulated for 3 μs (Fig. 2a), and the 2-D root mean squared deviation (RMSD) was measured across 5 individual trajectories revealing heterogeneous sampling across trajectories independent of initial structure (Supplementary Fig. 2a-b). We performed a clustering analysis using a 0.4 nm cutoff on each 15 μS ensemble by concatenating the 5 simulations to extract the top population sampled per peptide. We observe that this population represents only 33.5% of the sampled states for P301S R2R3_295-311_ while representing 46.2%, 64.2%, and 70.1% of states for WT R1R3_263-311ΔR2_, P270S R1R3_263-311ΔR2_, and WT R2R3_295-311_, respectively (Supplementary Fig. 2c). Using the mean structure of the top cluster as the reference (Fig. 2i), we calculated the RMSD to each peptide per trajectory to assess the effect of the initial structure on ensemble diversity (Fig. 2j). From these distributions we observe a distinction between the 3R and 4R derived peptides. WT R1R3_263-311ΔR2_ samples conformations below the 0.4 nm cutoff in trajectories T1 and T4, while the mutant P270S R1R3_263-311ΔR2_ samples the top cluster regardless of starting structure. We see opposing differences in the 4R peptides; WT R2R3_295-311_ sampled conformations below the cutoff in the trajectories started from expanded structures (T3-T5), while P301S R2R3_295-311_ only samples the top cluster structure in T1. These differences suggest that the 5 distinct N-terminal residues can modulate the sampling of conformations between the two isoforms and may reveal properties that distinguish the aggregation-prone state for the P301S R2R3_295-311_.

### Tau Peptide ensembles sample structures and interactions observed in vitro

To assess the sampling of conformations and interactions observed in our MD ensembles, we relate these features to the in vitro structural analysis by solution NMR spectroscopy and interactions probed by cross-linking mass spectrometry (XL-MS). First, we used an ensemble chemical shift predictor PPM^38^ to determine the theoretical chemical shifts from the MD ensembles of each peptide. We estimated the neighbor corrected structural propensity using the ncSPC software^41^ as done for the experimental chemical shifts (Supplementary Fig. 2d-e). We observe oversampling of the extended structure for the fragment, as the predicted chemical shifts from our MD ensemble all have negative deviations relative to the NMR chemical shifts (Supplemental Table 1). The neighbor corrected secondary structure propensities at the residues PGGG/SGGG have ncSP values closer to zero, corresponding with the turn region observed in the in vitro measurements (Fig. 1d and Supplementary Fig. 2d-e). For the N-terminal residues (263-269; **TE**N**L**KH**Q** and 295-300; **D**N**I**KH**V**), comparing the average ncSP values, we observe that the MD ensembles over sample extended structures by 36%, 37%, 52%, and 64% for WT R2R3_295-311_, P301S R2R3_295-311_, WT R1R3_263-311ΔR2_, and P270S R1R3_263-311ΔR2_, respectively. Looking at the C-terminal amyloid motif (306-310; VQIVY), our MD ensembles have approximately 40-67% higher extended structure propensity (average ncSP: −0.59 to −0.71) compared to that observed in the NMR experiments for both WT peptides (average ncSP: −0.04), the mutant peptides (average ncSP: −0.13) and this fragment from tauRD (bmrb 19253^23^, average ncSP: −0.08) and tau 225-324 (bmrb 50129^43^, average ncSP: −0.15). We note however that the negative ncSP values derived from our MD simulations (Supplementary Fig. 2d-e) correlate better with those observed for fragment 294-311 of tauRD and tau225-324 (Supplementary Fig. 1k,l), indicating that the structures sampled during the simulations are better represented in the NMR experiments peformed previously with the longer tau fragments.

To determine the residue-specific differences between the MD ensemble and the NMR experiments, we calculated the pairwise distances between all the hydrogens that could yield observable NOEs in our experiment. We weighted the distances using the 1/r^6^ dependence of the NOE cross-peak peak intensities (Supplementary Fig. 2f). The distance threshold that would be expected to produce an observable NOE cross peak for a peptide of this size is below 4Å (Supplementary Fig. 2f, red-yellow). This estimation shows sparse interactions between residues in the MD ensemble that would produce weak NOE cross-peaks (dist. 3.5-4 Å). The interactions correspond to the residues spanning the two sides of the observed beta-hairpin conformations. In our NOESY experiments we did not observe such medium- or long-range NOEs, but this is not surprising because the chemical shift analysis already indicated that more folded structures are more populated in the MD simulations than in the NMR experiments. Therefore, our ensembles represent the more compacted population of conformations observed in vitro, and the more expanded/random coil states are under-sampled. However, we observe a consistent distinction between the structural ensemble for aggregation-prone P301S R2R3_295-311_ and the three aggregation-resistant peptides. This data suggests that the MD ensembles still provide atomistic information about how changes in sequence alter the folding and stability of the collapsed conformations.

We next compared the MD ensembles to a recently published cryo-EM structure of tau fibrils isolated from tauopathy mice encoding P301S tau^39^. The structural comparison highlights that the ensembles containing a proline to serine mutation (P270S R1R3_263-311ΔR2_ and P301S R2R3_295-311_) have large populations compatible with the fibril conformation of this turn. Interestingly, the P301S R2R3_295-311_ has a bimodal RMSD distribution, suggesting a fraction of the ensemble is compatible with the fibrillar fold (Supplementary Fig. 2g). Lastly, to probe intramolecular interactions in vitro, we used a disuccinimidyl suberate (DSS) cross-linker to assess the accessibility and connectivity of pairs of lysines present in each of the four peptides (Supplementary Fig. 2h. top). Peptide samples were reacted with DSS, quenched, and analyzed by mass spectrometry. We evaluated populations of unmodified, single-modified (mono-links) and intra-molecular cross-links (i.e., loop-links). Data is interpreted as averages of 4 independent replicate reactions. Because poor fragmentation of the peptides prevented positional assignments, we only interpret the abundance of these three populations as estimated by peak area (Supplementary Fig. 4h). Interestingly, given the spacing of the lysines in the 4R-derived peptide sequences, we initially assumed that the lysines in the 4R peptides would be more accessible, leading to higher frequencies of mono-links compared to the 3R-derived peptide sequences. We find that the WT R2R3_295-311_ cross-link data trends similarly to the WT R1R3_263-311ΔR2_ peptide, and P270S R1R3_263-311ΔR2_ has the lowest proportion of single modifications (mono-links), yet we see higher modifications for the P301S R2R3_295-311_. We observe that all four peptides form a cross-link between pairs of lysines at a high frequency, with the P301S R2R3_295-311_ having the lowest frequency. Additionally, we see an increase in the amounts of unmodified peptides in P301S R2R3_295-311_ relative to the other peptides. This data hints at changes in the accessibility of the lysines (Supplementary Fig. 4h; K294, K298, and K311) with the addition of the proline to serine mutation that is distinct for the R2R3_295-311_ fragment. These data independently confirm changes in solvent accessibility of the fragments that differentiate aggregation-resistant from aggregation-prone sequences. Thus, the distinction in structural propensity and interaction potential with the amyloid motif in these fragments correlates with the aggregation propensity observed for these peptides in vitro.

### Intramolecular interactions involving non-polar contacts differentiate aggregation propensity.

**Figure 3.**
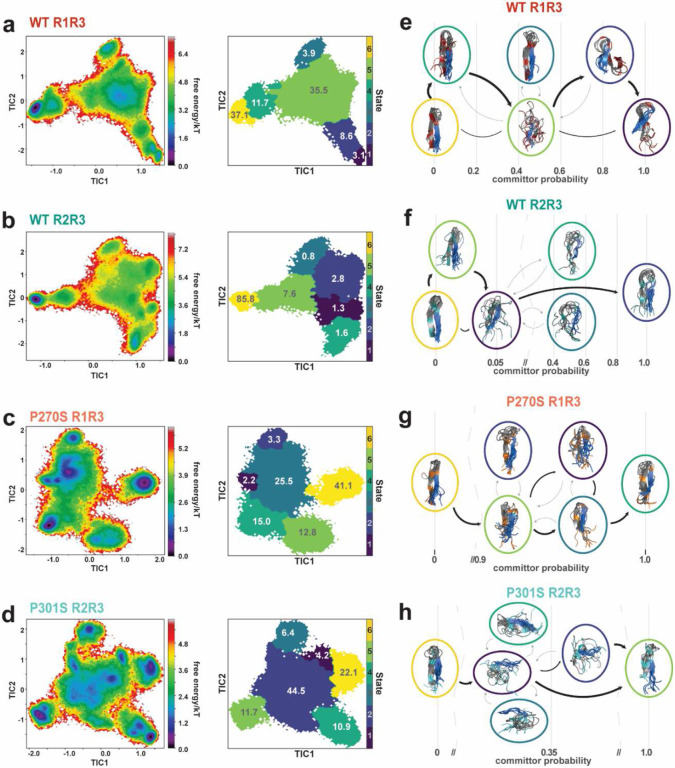
Unfolding transitions of tau fragments uncover rules that prevent aggregation. **a-d.** Free Energy landscape mapped onto the first two TICA components calculated form MD ensemble MSM for 15μs of simulation for WT R1R3_263-311ΔR2_ (a), WT R2R3_295-311_ (b), P270S R1R3_263-311ΔR2_ (c), and P301S R2R3_295-311_ (d). Data colored based on normalized free energy from black to red (left). MSM metastable state map, states 1-6 mapped from purple to yellow on free energy landscape (right). **e-h.** Transition pathway networks mapped on x-axis based on their committor probability for WT R1R3_263-311ΔR2_ (e), WT R2R3_295-311_ (f), P270S R1R3_263-311ΔR2_ (g), and P301S R2R3_295-311_ (h). The first 10 structures per state are shown in cartoon representation colored as in Figure 2h. Each state circle is colored based on the state map (a-d, right). Black arrows depict net flux sized based on pathway probability and gray arrows depict unproductive gross flux.

We used pyEMMA^45^ to build a Markov State Model (MSM) for each peptide to interpret folding transitions within the MD ensembles. We used the minimum pairwise distance between residues 1-8 against amyloid motif residues 12-18 as the discretization features. Estimating the implied timescale and comparing VAMP2 scores across different feature selections were used for model parameter selection (Supplementary Fig. 3a, b). The final MSM model was built using the k-means algorithm with 200 cluster centers and a maximum convergence time of 20 ns (200 steps), and the number of metastable states was determined to be 6 according to the PCCA+ algorithm. We validated the model parameters using a CK test for each peptide (Supplementary Fig. 3c). Using the validated MSM model, we projected a free energy surface onto the first two time-lagged independent component analysis (tICA) values and mapped the 6 states for each peptide (Fig. 3a-d). We calculated the pairwise distance of specific interactions (Supplementary Fig. 3e) to visualize the diversity of interactions sampled in each state (Supplementary Fig. 3f-j). We observe that the ‘PGGG’ turn-containing peptides (WT R1R3_295-311ΔR2_ and WT R2R3_295-311_) have a single defined local minimum (Fig. 3a, b; state 6, yellow) that corresponds to a beta-hairpin conformation. We assessed the distance distribution of specific interactions by the distance between the N- and C-termini and the distance between the left and right side of the fragment (mid-N-term to mid-C-term), as well as an extended amyloid motif (Supplementary Fig. 3e). Yet, we observe differences in the proportions of this state. WT R2R3_295-311_ state 6 accounts for 86% of the population compared to 37% for WT R1R3_295-311ΔR2_ (Fig. a, b b). This difference may stem from interactions mediated by the additional non-polar residue in the 4R-derived peptide (V300/Q269). Nonetheless, the mutant peptides containing the ‘SGGG’ motif had multiple low-energy states. The state that corresponds to the beta-hairpin fold, with similar distance properties as the ‘PGGG’ turn-containing peptides, in P270S R1R3_295-311ΔR2_ (Fig. 3c; state 6, yellow) accounted for 41% of the ensemble; however, for the aggregation-prone P301S R2R3_295-311_ this state (Fig. 3d; state 4, teal) only accounted for 11% of the ensemble (Supplementary Fig. 3f-h). Examining the remaining states, we observe that all four peptides sample a hairpin structure with changes in the register between the N- and C-terminal strands. To assess the change in the register with the positioning of the turn, we computed the ratio between the distance of residue K274/S305 to residue L276/I297 and residue Q269/V300, where a beta-turn centered at the ‘PGGG’ motif would result in a ratio close to 1, while a left-shifted turn would have a ratio <0.75, and a right-shifted turn would be >1 (Supplementary Fig. 3e,j). We observe that states 1-3 of WT R1R3_295-311ΔR2_ and 1-4 WT R2R3_295-311_ have this shifted register but represent a lower proportion of the ensembles. For P270S R1R3_295-311ΔR2_, we see that all the states exhibit this left-shifted register, but states 1, 3, 4, and 5 also have increased termini and left-right distances, suggesting these are more unfolded states (Supplementary Fig. 3f, g, j). A different pattern is observed for the aggregation-prone P301S R2R3_295-311_ peptide; the local minima, state 6, is consistent with the register shifted hairpin-like structure, but states 1-3 have a turn ratio close to 1 coupled with a collapsed N-terminal sequence (Supplementary Fig. 3i), and high left-right distances (Supplementary Fig. 3g); and state 5 shows a unique hairpin-like structure with a positive turn ratio corresponding to a right-shifted turn (Supplementary Fig. 3j). These four states with shorter N-terminal distances result from more collapsed conformations of the N-terminal segment and a more extended ‘SGGG’ motif. The addition of the non-polar residue V300 increases interactions with I297, stabilizing these conformations. These states are compatible with the distinct secondary structure propensity observed for the P301S R2R3_295-311_ peptide NMR. Therefore, these states may represent a larger proportion of the ensemble in the solution than our sampling suggests.

For transitions between states, we determined the mean first passage time (Supplementary Fig. 3d) and calculated the transition pathway between the lowest energy state (state 6) and the most divergent state for each peptide (Fig. 3e-h). We mapped the transition pathways based on the committor probability (the probability that each state is visited on-pathway to the end state) and visualized the flux through each state (Supplementary Table 2). For WT R1R3_295-311ΔR2_, the flux between state 6 and state 1 traverse states 4 to 5 to 2, in the top pathway accounting for 91% of the flux, revealing that the beta-hairpin state must sample several conformations, including the unfolded state, 5, to shift in register and reach state 1 (Fig. 3e, Supplementary Table 2). In the WT R2R3_295-311_ ensemble, the flux between state 6 and state 2 traverses states 5 to 1 and accounts for 78% of the flux1 (Fig. 3f, Supplementary Table 2); however, the committor probability of reaching these first states is less than 0.05, revealing that while the transitions between states 6 and 5 are fast (Supplementary Fig. 3d, MFPT <1 ns), few of these transitions result in flux to the divergent state 2 (Supplementary Fig. 3d, MFPT 5 to 2 = 27 ns). We also see unproductive transitions to states 3 and 4, and transitions from state 6 to these are much slower timescales (Supplementary Fig. 3d, MFPT 6 to 3,4 = 44, 42 ns). For the P270S R1R3_295-311ΔR2_ peptide, we see the opposite trend; the flux traverses 6 to 5 to 3 to 2, accounting for 67% of the flux, but all states have a very high committor probability >0.99 1 (Fig. 3g, Supplementary Table 2), so while transitions out of state 6 are relatively slow (Supplementary Fig. 3d, MFPT 10-28 ns), if any other state is reached there are fast transitions into state 4 (MFTP <1.5). The P301S R2R3_295-311_ ensemble top pathway traverses 6 to 1 to 2 to 5, accounting for 76% of the flux 1 (Fig. 3h, Supplementary Table 2); this pathway is distinct from the other peptides as the low energy state (6) is the left-register hairpin and the divergent state (5) is the unique right-register hairpin. Furthermore, the transitions between all states are relatively fast compared to the other ensembles (Supplementary Fig. 3d, MFPT<10 ns overall), resulting from the lower energy barriers between states. The on-pathway states (1 and 2) are more unfolded, and transitions to these states are fast (Supplementary Fig. 3d, State 2 MFPT <1 ns). Interestingly, the two off-pathway states illustrate the two divergent conformations of the peptide: state 4, which samples the beta-hairpin conformation, and state 3, which samples a collapsed N-terminus. These two folding transitions may delineate the pathways that inhibit or promote aggregation. These observations highlight the unique sampling of local conformations by the 4R-derived mutant peptide, consistent with this fragment's unique aggregation behavior. Moreover, we interpret that the first transition in P301S R2R3_295-311_ towards amyloid formation is mediated by higher accessibility of the amyloid motif, which may be dictated by a preference for local interactions between the two residues at the N-terminus.

### Ensemble-based design of 4R tauRD mutants reduces tau aggregation propensity.

**Figure 4.**
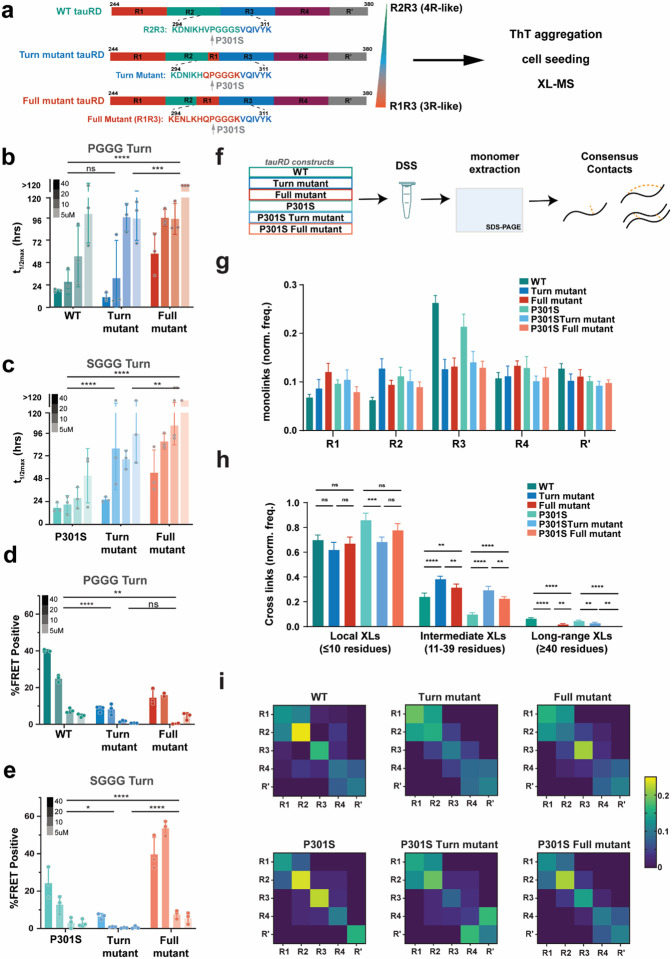
Designed tau sequences reduce aggregation of tau in vitro. **a.** Cartoon schematic of 4R tauRD and designed Turn mutant tauRD and Full Mutant tauRD. Repeat domains colored as in Figure 1, sequence inset of designed mutations below each schematic with residues colored based on repeat. **b-c.** ThT fluorescence aggregation assay of tauRD constructs induced with 2X heparin carried out at 4 concentrations: 5, 10, 20, 40 μM (light to dark) for ‘PGGG’ containing constructs WT tauRD (green), Turn mutant tauRD (blue), and Full mutant tauRD (red) in b, and ‘SGGG’ containing constructs P301S tauRD (cyan), P301S Turn mutant tauRD (sky blue), and P301S Full mutant tauRD (orange) in c. Data is shown as the t_1/2max_ fit to each aggregation curve. The data is plotted as an average with a standard deviation, significance calculated from column effects using 2way ANOVA (*=p < 0.05, **=p < 0.01, ***=p < 0.001, ****=p < 0.0001). **d-e.** Percent FRET positivity from lipofectamine-mediated seeding of pelleted fibrils form ThT-endpoints shown in b and c. FRET (tauRD_P301S-mClover3/tauRD_P301S-mCerulean3) was measured by flow cytometry on n = 3 biological replicates of at least 10,000 cells per condition of tauRD ‘PGGG’ and tauRD ‘SGGG’ constructs colored as in b-c. Data are shown as averages with standard deviation, significance calculated as in b-c. **f.** Schematic of sample preparation for XL-MS workflow. Tau constructs: WT tauRD, Turn mutant, Full mutant, P301S, P301S turn mutant, and P301S Full mutant (colored as in b-c); were cross-linked with disuccinimidyl suberate (DSS) then ran on an SDS-PAGE gel for monomer extraction, digestion, and purification to then be processed through our XLMS workflow for identification of consensus mono-links, and loop-link/cross-links. **g.** Quantification of identified mono-links grouped by repeat: R1 (244-274), R2 (275-305), R3 (306-336), R4 (337-368), and R’ (369-380); for tauRD constructs (colored as in f). Data is shown as the sum of the normalized mean and standard deviation across 5 replicated of the nseen for each construct. **h**. Quantification of local (< 10 residues), intermediate (11-39 residues) and long-range loop-links/cross-links (> 40 residues), by sample (colored as in f). Data is plotted as an average with a standard deviation, significance calculated one way ANOVA (*=p < 0.05, **=p < 0.01, ***=p < 0.001, ****=p < 0.0001). **i**. Heatmap of distribution of loop-links/cross-links for each construct grouped by repeat as in g. Data is shown as the average normalized frequency of nseen for each construct colored from navy to yellow.

To design aggregation-resistant 4R tauRD, we based our strategy on the sequence differences from the 3R isoform that can inhibit the aggregation of the VQIVYK amyloid motif and incorporated them in a 4R context. The first stabilizing construct uses the turn from R1 with the V300Q and S305K mutations (Fig. 4a; tauRD Turn mutant). The second mutant incorporates the full R1 element with four mutations: D295E, I297L, V300Q, and S305K (Fig. 4a; tauRD Full mutant). In addition to testing the constructs with the wild-type proline (i.e., ‘PGGG’ motif), we also tested our designs in the context of the P301S mutation (i.e., ‘SGGG’ motif). For all 6 constructs, we tested how the mutations influence aggregation propensity in vitro and in cells and identified differences in folding and interactions using high-resolution XL-MS (Fig. 4a). We evaluated the aggregation propensity of the constructs in a ThT fluorescence aggregation assay in a concentration series induced with heparin and fit the resultant curves to estimate a t_1/2max_ (Supplementary Fig. 4a-f, 40 μM, 20 μM, 10 μM, and 5 μM). We observe the minimal tauRD Turn mutant in a ‘PGGG’ context aggregates with similar rates to WT tauRD (Fig. 4b and Supplementary Fig. 4a, b; 40 μM/5 μM t_1/2max_ Turn mutant: 11.1±5.8 h/97.0±24 h; WT: 18.2±1.5 h/102.1±31.8 h). By contrast, the tauRD Full mutant with a ‘PGGG’ turn yields t_1/2max_ values that are 2- and 3-fold slower than WT tauRD (Fig. 4b and Supplementary Fig. 4c; 40 μM/5 μM t_1/2max_ Full mutant: 58.6±22.7 h/ >130 h). Interestingly, the engineered Turn mutants in the context of the ‘SGGG’ turn significantly slow the aggregation kinetics compared to the P301S tauRD (Fig. 4c and Supplementary Fig. 4d-f). The P301S tauRD Turn mutant aggregated between 2- and 4-fold more slowly compared to P301S tauRD (40 μM/5 μM t_1/2max_ P301S: 17.9±5.4 h/52.4±29.2 h; P301S Turn mutant: 26.7±2.9 h/97.7±31.7 h) while the P301S Full mutant further reduced aggregation leading to t_1/2max_ values between 3- and 5-fold slower than P301S tauRD (Fig. 4c and Supplementary Fig. 4d-f, 40 μM/5μM t_1/2max_ P301S Full mutant: 55.6±24.5 h/>130 h). TEM confirmed the presence of fibrils at the endpoint of the aggregation reactions for the 10 μM and 40 μM reactions (Supplementary Fig. 4g, h). At 40 μM, we observe defined fibrils for the WT tauRD constructs and P301S, P301S Turn mutant, and P301S Full mutant (Supplementary Fig. 4h). By contrast, for the Turn and Full mutants, we can only detect short fibrils and amorphous aggregates (Supplementary Fig. 4g). However, the largest differences are seen at the 10 μM concentration where the P301S construct forms long fibrils. Yet, the P301S Turn mutant only forms a few short fibrils, while WT, Turn mutant, and P301S Full mutant now only form short fibrils and amorphous aggregates, and the Full mutant had no visible aggregates or fibrils.

We next tested the seeding activity of the in vitro aggregation reactions in cells using a tau biosensor system in HEK 293T cells^46^. This cell-based tau seeding system detects tau aggregates with high sensitivity and specificity by converting the soluble tau fused to CFP and YFP tags into aggregates that can be detected via FRET^46^. Endpoints of the in vitro ThT aggregation reactions were sonicated and transfected into tau biosensor cells. The amount of seeding was quantified using flow cytometry using a gating strategy to estimate the percentage of cells that were FRET positive (Supplementary Fig. 4i). Compared to the WT tauRD fibril samples, we observe on average a 5- and 6-fold reduction in seeding for cells treated with the Turn mutant and the Full mutant samples across the four concentrations, respectively (Fig. 4d). Similarly, for the samples that contained the P301S mutation, we observed on average an 8-fold reduction in seeding for the P301S Turn mutant sample across the concentration series (Fig. 4e). Interestingly, for the P301S Full mutant we observe on average a 2-fold increase in seeding compared to P301S tauRD (Fig. 4g). We speculate that this may be due to producing more efficient seeds from the smaller assemblies observed in the P301S Full mutant (Supplementary Fig. 4h). Cumulatively, we observe that introduction of the 3R-like mutations into tauRD in proximity to the VQVIYK amyloid motif on average reduces aggregation propensity of tau and that the changes in aggregation kinetics consistent with the changes in seeding capacity measured in cells.

### Balance of intermediate and long-range contacts correlates with inhibition of tau aggregation.

To understand how tauRD conformation changes in response to the designed mutations, we turned to XL-MS. This approach enables capturing interactions in complexes to define architectures of assemblies^47-49^. More recently, our lab has established ways to probe changes in the conformation of IDPs using XL-MS^12,13,50^. We have used a panel of cross-linkers to interrogate how electrostatic changes dictate the misfolding steps of tau and other amyloid-forming proteins^12,51^. Previously, we have used this approach to probe the effect of P301S on tauRD dynamics, uncovering that this mutant is more sensitive to unfolding and, thus, more competent to self-associate^13^. Here, we extend this approach to understand how the designed mutations change the distribution of local contacts in proximity to the mutation and longer-range contacts to relate these changes to the reduction of tau aggregation. The 6 purified tauRD constructs were reacted with the lysine-reactive DSS (DSS, spacer arm 11.4A) cross-linker and then quenched. The cross-linked proteins were resolved on an SDS-PAGE gel, and the monomeric bands were excised and proteolyzed^51,52^. The samples were analyzed by mass spectrometry, and the data was processed using the xQuest pipeline^53^. Each sample was processed as 5 independent replicates, and only consensus contacts with high scores and low FDRs were considered for further analysis (Fig. 4f). We first compared the frequency of mono-links, defined as DSS modifications on a single lysine. The normalized frequency of mono-link modifications mapped onto each repeat domain is similar except for WT and P301S, where there is a significant increase in repeat 3 despite a similar frequency of modification across the rest of the sequence (Fig. 4g and Supplementary Fig. 5a). We interpret this difference as an increased reactivity of lysine residues in repeat 3, which contains the VQIVYK motif, and therefore less accessibility of this region for all designed constructs independent of ‘PGGG’ or ‘SGGG’ (Supplementary Fig. 5a). We next interpret the loop-links/cross-links in these samples, defined as intramolecular covalent contacts between pairs of lysines located on two peptides or within a peptide, reported on contact maps, showing unique cross-link pair colored by the normalized frequency detected across 5 replicates (Supplementary Fig. 5b, c). To help guide the interpretation of the cross-links, we group them by contact order, separating the cross-linked positions to help distinguish contacts within repeats (local; ≤ 10 residues), between adjacent repeats (intermediate; 11-39 residues), and long-range interactions (≥ 40 residues). First, a comparison of the WT and P301S tauRD XL-MS analysis reaffirms our previous findings that the P301S mutation appears to destabilize intermediate and long-range contacts within the repeat domain of tau while the relative frequency of local contacts increases (Fig. 4h). For the PGGG-based Turn and Full mutants’ comparison, the WT tauRD profile shows a significant increase in intermediate contacts but a loss of a small fraction of long-range contacts (Fig. 4h and Supplementary Fig. 5b). A similar change in pattern is observed for the SGGG-based Turn and Full mutants when compared to the P301S tauRD where the designed mutants yield a boost in intermediate contacts (Fig. 4h; and Supplementary Fig. 5c). Mapping these cross-links by repeat domain (i.e., R1-R4) we observe that the shift from long-range to intermediate interactions corresponds to a shift from N-terminal interactions in Repeat 1-2 in WT and P301S to more central interactions in repeats 2-4 for the designed constructs (Fig. 4i). To assess this shift in more detail, we used a hierarchical clustering approach to build a dendrogram that relates the similarity of the cross-linked patterns and their abundance across 6 constructs. We find that local contacts cluster separately from long-range, and intermediate contacts cluster into two groups (Supplementary Fig. 5d). The cross-links cluster into four groups: 1) high-frequency local cross-links (yellow), 2) intermediate contacts absent in P301S (green), 3) mostly long-range contacts with low abundance identified in WT (blue), and 4) intermediate contacts unique to the designed construct with both the ‘PGGG’ and ‘SGGG’ turn (purple). Finally, we relate the abundance of contacts to K311 within the VQIVYK amyloid motif and find that in both the ‘PGGG’ and ‘SGGG’ designed constructs, we have more unique interactions at this position. We also see interactions to our fragment of interest, position K280/K281, and the K311 not identified in either WT or P301S tauRD (Supplementary Fig. 5e). Across the designed constructs, the most significant changes localize to intermediate-range interaction between repeats 2, 3, and 4. Comparing these constructs' aggregation behavior, we see that these increased interactions correlate with reduced aggregation propensity. Overall, we find that a balance of long and intermediate contacts is helpful for aggregation prevention, and the loss of these increases the unfolding and exposure of the amyloid motif.

### Aggregation-reducing tau mutations maintain microtubule-binding activity.

**Figure 5.**
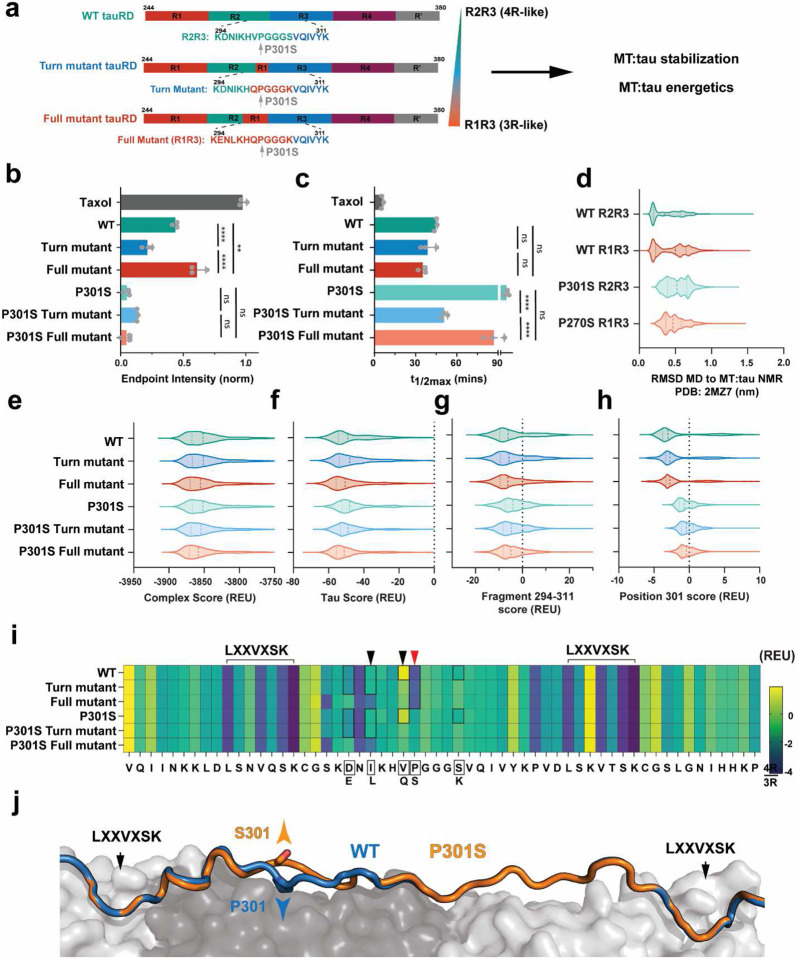
Designed tau sequences retain MT activity. **a.** Cartoon schematic of 4R tauRD and designed Turn mutant tauRD and Full Mutant tauRD as in Figure 3. **b.** Microtubule polymerization assay endpoint intensity of tauRD constructs at 10 μM for ‘PGGG’ containing constructs WT tauRD (green), Turn Mutant TauRD (blue), Full mutant tauRD (red), and ‘SGGG’ containing constructs P301S tauRD (cyan), P301S Turn mutant tauRD (sky blue), and P301S Full mutant tauRD (orange). Data is shown as intensity mean and standard deviation, significance calculated from column effects using 2way ANOVA (*=p<0.05, **=p<0.01, ***=p<0.001, ****=p<0.0001). **c.** Microtubule polymerization assay t_1/2max_ fit to each polymerization curve plotted as an average and standard deviation, significance calculated as in b. **d.** RMSD between tau fragment 295-311 bound to MTs (bmrb 25475) to 15 μs MD ensembles for peptides: WT R2R3_295-311_ (green), WT R1R3_263-311ΔR2_ (red), P301S R2R3_295-311_ (cyan), and P270S R1R3_263-311ΔR2_ (orange). The data is shown as violin plots with median and 25/75% quartiles labeled in dashed lines. **e-h.** Rosetta energy scores from 2500 models for the full MT:tau complex (e), tau 275-331 (f), built fragment 294-311 (g), and residue 301 (h). The data is shown as violin plots with median and 25/75% quartiles labeled in dashes lines. **i.** Residue energy for tau 275-331 from MT:tau modeled complex for designed constructs. Energies shown as mean REU of 2,000 structures colored from High energy to low energy (yellow to navy). Fragment sequence below with boxed residues signifying 4R tauRD sequence. Labels above heatmap denote MT:MAP anchor motifs. Red arrows denote position 301, and blank arrows denote position 297 and 300 that have lower energy for designed mutations. **j.** Lowest energy structure of modeled sequence for WT tau275-331 (blue, cartoon) and P301S tau275-331 (orange, cartoon) in complex with MT (gray, surface). Black labels denoting MT:MAP anchor points, position 301 labeled for WT pointing down (blue, sticks) and P301S pointing up (orange, sticks).

The endogenous function of tau consists of stabilizing microtubules (MT)^21^. To determine how our engineered tau constructs alter this function, we performed a fluorescent MT polymerization assay coupled with molecular modeling of the MT:tau complex. Purified tubulin dimer was incubated with each tau construct. The rate and extent of polymerization relative to tubulin alone were determined by fitting the resultant curves (Supplementary Fig. 6a, b). For the ‘PGGG’ turn constructs, we observe that the Turn mutant tauRD polymerization amplitude was significantly reduced by 1.5-fold. In contrast, the Full mutant tauRD had increased polymerization by 1.5-fold relative to WT tauRD (Fig. 5b and Supplementary Fig. 6a). Interestingly, all three constructs had no significant difference in the t_1/2max_ time to endpoint polymerization (Fig. 5c and Supplementary Fig. 6a; 44.8±1.1 mins WT, 38.7±6.0 mins Turn mutant, 35.3±2.9 mins Full mutant). Mutation of the proline to serine at position 301 has previously been shown to alter binding to MT^24,54^. Consistent with this observation, we see a nearly 10-fold reduction of polymerization for P301S tauRD compared to WT tauRD (Fig. 5b and Supplementary Fig. 6b). Introducing the Turn-only or Full mutations in the P301S background does not significantly recover MT binding (Fig. 5b and Supplementary Fig. 6b). However, the above baseline polymerization of the P301S Turn mutant had a t_1/2max_ time approaching that of the t_1/2max_ determined for the ‘PGGG’ containing tauRDs (Fig. 5c and Supplementary Fig. 6b). Our data highlight that the P301S mutations significantly alter MT binding and that neither Turn nor Full mutations can recover this loss. In the context of the ‘PGGG’ turns, the Full mutant appears to increase the extent of binding without changing the rate of polymerization. We next wondered whether these data could be interpreted in the context of available MT:tau structural data.

### Modeling the MT:tau complex reveals key stabilizing interactions.

Local structure in tau has been implicated in its binding to MTs, with the addition of the P301S mutation resulting in almost complete loss of binding to tau^54^. Available structural models of tau in the context of microtubules include tau in a fully extended conformation determined by cryo-EM^22^ and a more compacted conformation visualized by NMR^55^ that may be indicative of the conformation of tau as it transitions from the unbound to the MT-bound state. We compared our MD ensembles to the MT-bound conformation of tau determined by NMR (PDB ID 2MZ7)^56^. The RMSD partitions by turn sequence composition, ‘PGGG’ vs. ‘SGGG,’ suggesting that the proline-containing turn may play an important role for tau to access the conformation needed for binding to MTs (Fig. 5d). To explore how these turn sequence changes may affect the bound state of the MT:tau complex, we modeled the interaction energetics of different tau constructs bound to MTs using an available cryo-EM structure (EMDB-7771). First, we built WT and mutant 4R tauRD into the available 4xR2 tau from the cryo-EM model (PDB ID 6CVN) using a threading protocol to rebuild the missing sequence and produced 2,500 models constrained by the initial template. These models were then trimmed to contain only repeats 2 and 3 (275-331) and minimized in the context of tubulin α/β/α trimer, and the energetics and conformational heterogeneity were compared for all 6 constructs (Supplementary Fig. 6c-e). The 10 lowest energy complexes show a diverse sampling of loop positions for the turn region (Supplementary Fig. 6c-e), with higher variability in sequences containing the ‘SGGG’ motif (Supplementary Fig. 6c, e). We compared the energetics of our models by scoring the MT:tau complex and found only modest energy differences between WT and P301S (Fig. 5e). We then focused on the scores of tau only, the 294-311 tau fragment, and finally only the 301 position. As we hone in on the loop region, we observe an increased difference in energetics between the ‘PGGG’ and ‘SGGG’ models, with the largest differences derived from the 301 position alone (Fig. 5f-h). Indeed, plotting the per residue energy contributions of the fragment, we find that the proline to serine yields the most dramatic shift (Fig. 5i, red arrow) and sits firmly between two well-established tubulin binding motifs (Fig. 5i, LXXVXSK motifs)^55,57^. The changes in the energy terms that dictate this difference are the burial of the proline ring (Supplementary Fig. 6f). While the serine has the potential to form an additional hydrogen bond, the energy contribution from that interaction does not overcome the increased van der Waals forces (Supplementary Fig. 6f). At the same time, the serine residue points away from the MT (Fig. 5j). Interestingly, we also find that valine to glutamine mutations at position 300 (from Turn mutant) as well as isoleucine to leucine mutations at position 297 (from Full mutant) have lower energy and contribute to complex stability (Fig. 5i, black arrows). This boost in stabilization may explain why the full mutant may yield higher MT binding compared to the WT tauRD (Fig. 5b). When comparing the available MT:tau structures, there is clear importance of the proline at position 301, suggesting not only an effect on the bound state energetics but the potential impact on the ability to transition into this state.

### Stabilizing mutations render tau less sensitive to exogenous seeds in cells.

**Figure 6.**
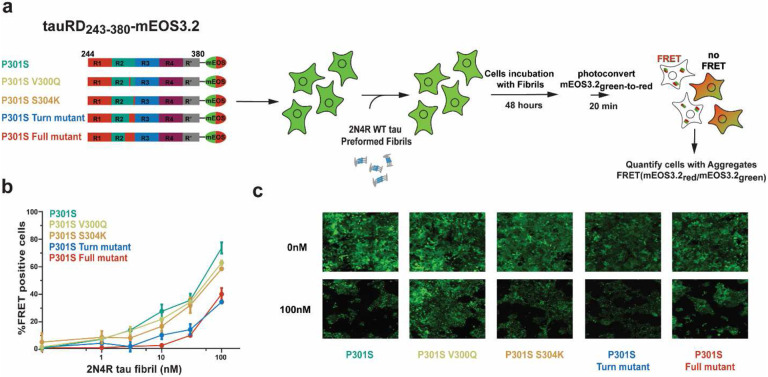
Designed tau sequences reduce tau aggregation in cells. **a.** Schematic of fibril propagation seeding workflow. Cartoon of 4R tauRD P301S designed construct with fusion to mEOS3.2, used for lentiviral transfection into HEK 293T cell and sorted for high expression, treated with lipofectamine mediated seeding from 2N4R WT tau preformed fibrils, followed by incubation time, photoconversion and analysis by percent FRET positivity as in Figure 3. **b.** Percent FRET positivity (FRET pairs: tauRD-mEOS3.2green/tauRD mEOS3.2red) was measured by flow cytometry on n = 3 biological replicates of at least 10,000 cells per fibril concentrations and expression construct. Data are shown as averages with standard deviation. **c.** Imaging of HEK 293T cells expressing designed constructs 48hrs after no treatment control and max treatment of 100 nM fibril concentration.

Finally, to evaluate whether our designed tau sequences influence response to pathogenic seeds, we developed a cellular assay leveraging mEOS3.2 fusions of tauRD harboring our designed mutants in the context of the pathogenic P301S mutation. To assess the behavior of these mutants, we employed a HEK293T cell model of tauRD aggregation leveraging the FRET-compatible photoconvertible mEOS3.2 system. We produced cell lines stably expressing P301S, P301S_V300Q, P301S_S305K, P301S Turn, and P301S Full mutants as tauRD fusions to mEOS3.2, which were sorted to maintain similar high expression levels across cell lines. The cells were seeded with a concentration series of recombinant tau fibrils, photoconverted, and then analyzed by flow cytometry (Fig. 6a). To ensure optimal FRET efficiency, the photoconversion time was determined empirically (Supplementary Fig. 7a). Then, we employed a gating strategy to observe the proportion of cells that were FRET-positive (Supplementary Fig 7b). As observed previously, P301S tauRD-mEOS3.2 yielded high percentages of cells, with 74±4% of FRET-positive cells seeded with 100 nM recombinant tau fibrils (Fig. 6b, green). The introduction of V300Q and S305K mutations alone reduced seeding 1.2-fold (Fig. 6b, yellow and orange) while combining these mutations in the Turn mutant (V300Q and S305K) reduced seeding activity nearly 2-fold (Fig. 6b, blue). Finally, introducing the Full mutant reduces seeding activity by 2-fold compared to the P301S tauRD control (Fig. 6b, red). Representative images of cells expressing P301S, P301S_V300Q, P301S_S305K, P301S Turn, and P301S Full mutants as tauRD seeded with 0 nM and 100 nM recombinant tau fibrils show reduction in puncta frequency (Fig. 6c). Our data supports the idea that modification of the turn residues is sufficient to reduce P301S tauRD sensitivity to pathogenic tau seeds in cells.

## DISCUSSION

The conversion of tau into insoluble aggregates is linked to many neurodegenerative diseases. Tau isoform conversion between 3R and 4R leads to different aggregation propensities; nevertheless, only minor sequence changes surrounding amyloidogenic motifs provide mechanistic hints toward tau aggregation regulation. Here, we use a combination of computational, biophysical, and cellular approaches on tau fragments and the intact repeat domain to test rationally designed tau sequences that uncover differences in 3R vs. 4R tau isoforms that slow their capacity to assembly into beta-sheet rich amyloids.

**Figure 7.**
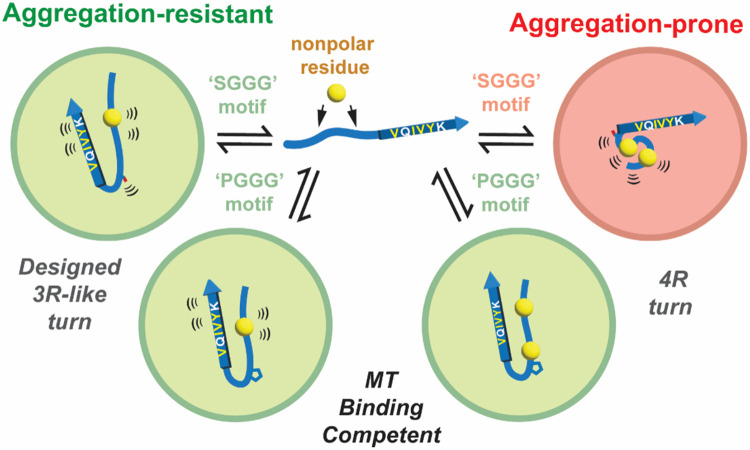
Model for regulation of local structure to reduce aggregation and retain biological activity. Interactions between the N-terminal nonpolar residues (yellow spheres) in the 4R and the designed 3R-like and the amyloid motif are stabilized by ‘PGGG’ motif and sample conformations compatible with MT binding to reduce overall capacity of tau to self-assemble (green circles, bottom). A mutant ‘SGGG’ turn in the 3R-like sequence destabilizes the nonpolar N-terminal interactions with the amyloid motif but overall, still samples turn conformations (green circle, left). By contrast, a mutant ‘SGGG’ turn in the 4R sequence unfolds the element shifting the two N-terminal nonpolar residues to self-associate releasing the amyloid motif thus promoting aggregation (red circle, right). Destabilization of the turn through the mutant ‘SGGG’ turn yields conformations less suitable for binding to MTs.

We find that the conserved ‘PGGG’ motif, coupled with the residues that flank it, plays a vital role in limiting the self-assembly of the amyloidogenic ^306^VQIVYK^311^ (Fig. 7). However, differences in the flanking sequences between 3R and 4R tau profoundly affect aggregation by changing the stability of interactions with ^306^VQIVYK^311^. Based on in vitro NMR and XL-MS and in silico MD ensemble measurements, we see sequence-based rearrangements of local interactions mediating the exposure of the amyloid motif. In 3R tau, the nonpolar residue in the N-terminus engages with the amyloidogenic motif to prevent aggregation even in the context of the aggregation-promoting proline to serine mutation (Fig. 7, left side). By contrast, the wild-type 4R fragment shows stable interactions between the two N-terminal nonpolar residues and the amyloid motif; just introducing the P301S mutation yields distinct conformations in which the N-terminal nonpolar residues preferentially cluster with themselves, thus exposing the amyloid motif to promote aggregation (Fig. 7, right side). Validating our observations based on peptides using the tau repeat domain, we confirm their reduced capacity to aggregate and the retention of the microtubule-stabilizing activity when containing a ‘PGGG’ motif but not in the mutant ‘SGGG’ sequences (Fig. 7). We identify a structural model for how the proline to serine mutations may alter binding to MTs – modeling of tau bound to MTs based on the available cryo-EM data suggests that the proline to serine mutant largely drives changes in the stability of the interaction. Together, our data suggest that it may be possible to tune tau aggregation by stabilizing local interactions with amyloidogenic motifs while maintaining biological activity.

The field has focused on understanding the pathological effects of endpoint aggregates to guide potential ways to diagnose or treat disease. How tau interacts with MTs has garnered less interest. It remains unclear what features of microtubule-associated proteins (MAPs) are required for binding to MTs. For many years, it has been proposed that the repeat domain of tau (also known as the Microtubule Binding Region, MTBR) was the minimal fragment of MAPT required to facilitate interactions with MTs. Yet, recent evidence shows that the proline-rich domain contributes to binding^58^. Our modeling data and a prior body of literature support that anchor points in MAPs, including MAPT, are utilized for binding to MTs^57^. For example, our data shows that the ‘LXXVXSK’ motif conserved across MAPs anchors tau at sites with defined spacing at the interface between α/β-tubulin heterodimers^55^. However, it has been unclear what the role of the conserved ‘PGGG’ turns has been in interaction with MTs. Pathogenic mutations in tau largely localize to the repeat domain, particularly within and near the conserved ‘PGGG’ turns^27^, consistent that they may destabilize MT binding. Most of these mutations are also linked to increasing aggregation propensity of tau^59,60^. This double-edged sword increases concentrations of cytosolic unbound tau with an increased aggregation capacity. New ways to engineer tau to reduce its aggregation propensity while maintaining biological activity are necessary.

Our data demonstrate that the substitution of four residues in proximity to the VQIVYK motif in tau to mimic the 3R localized sequence not only reduces 4R tau aggregation in both wild-type ‘PGGG’ or mutant ‘SGGG’ turn motif backgrounds but also maintains the ability of tau to stabilize MTs. Prior cryo-EM structural data on tau bound to MTs showed a fully extended conformation, highly disordered at the inter-repeat regions surrounding the ‘PGGG’ motif. By contrast, NMR studies showed a relatively collapsed conformation of this element bound to MTs, adopting a turn conformation around the ‘PGGG’ motif. Our analysis begins to explain how the wild-type proline at this position may be important for tau binding to MTs. In our analysis of the MT:tau interactions, the ‘PGGG’ and ‘SGGG’ do not appear to make important stabilizing interactions with the MT but rather alter the dynamics of this region, which indirectly perturbs the stability of neighboring binding sites. The difference in the ‘PGGG’ compared to ‘SGGG’ dynamics likely is born out of the ensemble state of tau in the unbound state and is consistent with our MD analysis. Suggesting that these conformations are sampled in solution and contribute to the burial of the proline ring on the surface of the MT with favorable energetics. Modeling of MT:tau also explains why the proline in the ‘PGGG’ motif enables better MT stabilization than the serine in ‘SGGG.’ These energetic changes suggest that the proline directly stabilizes interactions with the MTs and enables the more rapid formation of local structures consistent with an encounter complex. By contrast, the serine does not contribute energetically to the bound state and destabilizes conformations required to form an encounter complex. Thus, to fully understand how to improve tau function, we must consider the ensemble of tau in solution and its compatibility with the MT:tau encounter complex, the final interactions that must be formed with MTs, all the while considering how to prevent amyloid-motif dependent assembly into pathological states.

A deeper comparison of naturally occurring MAP sequences may offer clues for the design of tau sequences that retain MT binding but aggregate less. The *MAPT* gene is highly conserved across evolution^57^. Similarly, the related *MAP2* and *MAP4* genes are conserved but do not encode aggregation-prone sequences^57^. Comparison of *MAPT* to *MAP2/MAP4* reveals high similarity in many ways. The genes are processed similarly during splicing, producing both 3R and 4R isoforms of the sequence that retain binding to MTs. Only MAPT is linked to misfolding diseases despite related architectures and sequences, while the other MAPs are not. This similarity raises an interesting question: why are aggregation-promoting sequence features of the tau preserved in evolution? Indeed, in the MAP sequences, 2 of the 4 amino acids in the essential amyloid motif, **V**Q**I**VYK (bold are conserved and underlined are variable), are conserved across MAPs. Because this site is essential for splicing, and even codon changes can alter splicing, part of the amyloid motif may be preserved to retain efficient splicing. However, it is unclear why the latter part of the amyloid motif is conserved. By contrast, inference of sequence conservation in the context of MAPT bound to MTs shows that a subset of residues point away from the MT interface and largely coincide with variable positions across MAPs^57^. The local difference between the 3R and 4R isoforms in MAPT is also consistent with this variation; mutation of valine to glutamine at position 300 seems to benefit both MT binding and confer protection against aggregation by removing a nonpolar residue that alters the unfolding of the local sequence and interaction with the amyloid motif.

Analysis of disease-associated mutations in large repositories in the context of globular proteins (using AlphaFold models) shows that gain-of-function (GOF) and loss-of-function (LOF) have very different distributions on the protein structure^61^. LOF mutations are randomly distributed, but GOF mutations are clustered at sites that disrupt an important biological function^61^. If we apply this principle to MAPT, we find that pathogenic mutations largely localize to the repeat domain of MAPT and, more specifically, cluster at inter-repeat elements^27^, including specific mutations in the variable exon 10 of *MAPT* that are associated with altering splicing^29^. This defined clustering of mutations may suggest that a combination of LOF and GOF mechanisms is linked to pathological fibrilization and the loss of MT binding. Future experiments and analysis must more closely interrogate the relationship between the tau monomer ensemble in solution, MT binding states, and the early misfolding steps that lead to disease. Thus, more focus must be brought onto the effect of mutations on the multi-state problem of tau conformation in solution (aggregation-resistant), tau bound to MT (functional state), and tau misfolding towards pathological aggregates to begin to understand the origins of tau in function and disease.

We uncover how sequence changes alter the folding/unfolding of minimal elements in tau and discover that the combination of two nonpolar residues N-terminal to the amyloid motif combined with an ‘SGGG’ turn enables disengagement of the N-terminus from interaction with amyloid motifs. This release of the amyloid motif promotes VQIVYK-VQIVYK homotypic interactions consistent with the early interactions on pathway to fibrillar disease states^16^. The local unfolding extends to large changes in the interactions in full-length tau between the acidic N-terminus and the basic repeat domain encoding pathogenic mutations^12,51^. A recent study reaffirmed this idea, proposing that mutations in tau lead to a more unfolded ensemble that can rapidly nucleate aggregation^62^. We suspect these intermediate and long-range interactions may play an important role in forming the MT:tau encounter complex. Interestingly, XL-MS experiments on tauRD encoding mutations that convert the local sequence from 4R-like to 3R-like alter the frequency of global long-range contacts but increase medium-range contacts. This shift in frequency of interactions correlates with stabilization of the ‘PGGG’ turn to reduce aggregation and maintain or even increase MT activity. Future experiments may explore how the computational design of tau may lead to mutants that can replace wild-type MAPT to reduce disease but, importantly, may shed light on the biology that underlies MAPT function beyond its link to amyloid formation.

Our data supports that local structural rearrangements in tau surrounding amyloid motifs play important roles in the regulation of tau aggregation. The ability to adopt protective anti-aggregation conformations is coupled between ‘PGGG’ turn motifs and their upstream sequences to engage with the amyloid motifs. The design of sequences that stabilize these conformations and interactions offers new strategies to limit aggregation and maintain the biological activity of MAPT for binding to MTs. A deeper interpretation of the evolvability of tau to tune these two properties may be inferred from the evolutionary sequence history of MAPs. Future studies must test the biological consequences of designer MAPT sequences in animal models as ongoing therapeutic approaches to reduce tau levels may lead to unknown consequences in humans.

## METHODS

### Peptide synthesis and disaggregation

All peptides were synthesized as ordered by Genscript with N-terminal acetylation and C-terminal amidation modifications at >95% purity. Peptides were disaggregated as previously described^63^. In brief, peptides were resuspended in a 250 μL Trifluoroacetic acid (TFA) (Pierce) incubated at room temperature (RT) for 1-16 hours in a chemical fume hood, the peptide solution was dried under a stream of nitrogen gas, and then 100 μL of H_2_O was added, followed by flash freezing and immediately placed under vacuum to remove any residual solvents. The peptide residue was resuspended in 200 μL of H_2_O to determine the concentration and, with the addition of 10X PBS pH 7.4, adjusted with NaOH and H_2_O to achieve the desired pH and concentration.

### ThT fluorescence aggregation assays

Peptide concentrations were adjusted with 1X PBS (136.5 mM NaCl, 2.7 mM KCl, 10 mM Na_2_HPO_4_, 1.8 mM KH_2_PO_4_, pH 7.4) and 1 mM Dithiothreitol (DTT) to a desired concentration between 800 μM and 50 μM. Thioflavin T (ThT) was added to the samples at a final concentration of 25 μM. Wild-type or mutant 4R or 3R tauRD protein was diluted in 1X PBS with 10 mM DTT to the desired concentration of 40, 20, 10, and 5 μM. A 2X heparin concentration (Amsbio) was added to the tauRD protein. All samples were aliquoted at 45 μL per replicate in a 384-well clear bottom plate. All conditions were performed as three independent replicates at 37 °C. ThT kinetic scans were run every 30 min, with 10 seconds of orbital shaking before each read, on a Tecan Spark plate reader at 446 nm Ex (10 nm bandwidth) and 482 nm Em (10 nm bandwidth). Blank wells containing buffer and ThT were subtracted from experimental values. The data were plotted, and the *t*_1/2_ values were estimated using a non-linear regression model fitting in GraphPad Prism and reported as averages with a standard deviation.

### Peptide NMR and secondary structure calculation from chemical shifts

NMR spectra were acquired on Agilent DD2 spectrometers operating at 600 MHz at the UTSW Biomolecular NMR Facility. ^1^H-^1^H TOCSY spectra^64^ and ^1^H-^1^H NOESY spectra^64^ were acquired at 25 °C. Peptides were adjusted to 500 μM in 1X PBS, 1mM DTT with 10% D_2_O or 99.998% D_2_O, and the total acquisition time for ^1^H-^1^H TOCSY and ^1^H-^1^H NOESY spectra ranged from 8-11 hours. Five ^1^H-^1^H TOCSY spectra were collected in series for the aggregation-prone P301S R2R3_294-311_ peptide, each 10 hours long. All data were processed with NMRpipe^65^ and analyzed with CARA (http://cara.nmr.ch/doku.php) and SPARKY^66^. Secondary structure propensity was calculated from the neighbor-corrected secondary structure propensity calculator (ncSPC) from the HA chemical shift assignments. The calculator used the deviations from random coil chemical shifts to estimate the proportion of secondary structure per residue corrected by a specified neighbor window. For our analysis we used a neighbor residue window of 3^41,42^. The data were plotted in GraphPad Prism.

### Molecular dynamics simulations production

Molecular Dynamics simulations were performed using PLUMED patched GROMACS v2018.4. Each peptide was simulated in a dodecahedron box with SPC/E water starting from a fully extended conformation built-in Pymol for a total of 5 μs followed by 15 μs in TIP3 water with 150 mM NaCl ions. The initial dodecahedron simulation box was constructed using the extended peptide structure with a minimum 1.5 nm boundary condition. The AMBER99sb-ildn forcefield^44^ was used for all simulations. After an initial 1000 steepest descent steps of converged energy minimization, 10 ns of NVT and 20 ns of NPT (first 10 with Berendsen^67^ and the last 10 with Parrinello-Rahman^68^ barostats) equilibrations were performed. The subsequent production level trajectories are based on 5 fs time steps using hydrogen-only virtual sites^69^. Production level trajectories were obtained for an NPT ensemble with Parrinello-Rahman barostat and periodic boundary conditions with Particle Mesh Ewald (PME)^70^ summation for long-range electrostatics. A total of 20 μs trajectories were generated by producing five 1 μs simulations per peptide. These trajectories were analyzed using the GROMACS clustering function with a cluster cutoff of 0.54 nm. The cluster cutoff was chosen empirically to produce a maximum of 15 clusters for all four peptides. The center structure of the top 5 clusters was then used as the starting structure for simulations with Na+ and Cl− ions included in the solvent. These second stage simulations were performed with the same minimization, equilibration, and production workflow with only a few modifications. A dodecahedron simulation box was used, using the new starting structures with an increase in the minimum boundary cutoff to 1.6 nm. The same AMBER99sb-ildn forcefield^44^ was used with an update to a TIP3P water model with the inclusion of 150 mM ionic strength by inclusion of Na+ and Cl− ions. The energy minimization step was performed with the steepest decent algorithm to obtain a maximum force <1000.0 kJ/mol/nm. Equilibration was run aligned with the previous step. The subsequent production level trajectories were performed as the initial simulations, with an updated 2 fs timestep to account for the ionic solvent. A total of 60 μs trajectories were generated by producing five 3 μs trajectories per peptide. All simulations were done on UTSW’s BioHPC computing cluster.

### MD analysis: clustering, RMSD, and distance calculations

All analysis was done using GROMACS 5.0.4^70^. The analysis framework was done as previously described^63^. Specifically, minimum distance heatmaps in Fig. 2b-e were calculated based on the mean minimum distance using the ‘gmx mdmat ‘command. Backbone distance in Fig. 2f. was calculated using the ‘gmx pairdist’ command between the center of mass of backbone atoms for residues 1-6 (i.e., XXNXKH) vs. residues 12-18 (i.e., XVQIVYK) from the 5 μs of the initial replicate simulations. Data is shown as violin plots (e.g., density distributions) with mean and 25% and 75% quartile marked. For the second step of simulation the 5 independent trajectories were concatenated into a 15 μs ensemble and the 2-dimensional RMSD was calculated every 10 ns using the ‘gmx rms’ command. Clustering of MD ensembles was done using the ‘gmx cluster’ command. For the initial 5 μs simulations a cutoff of 0.54 nm was used based on producing less than 15 clusters for all peptides. For the second step 15 μs simulations a cutoff of 0.4 nm was used based on the RMSD deviations observed across the 2D RMSD comparisons. RMSD distributions per trajectory referenced to each peptide's Cluster 1 structure shown Fig. 2I were calculated for each 3uS of simulation and shown as violin plots. The 15 μs ensembles were also compared to published structured of tau, first the RMSD of the ensemble referenced to P301S fibril structure (PDB ID 8Q96) reported as a distribution, violin plot. Second, RMSD of the ensemble referenced to state 1 of the NMR structure of tau in the presence of α/β tubulin (PDB ID 2MZ7). All graphs were plotted directly from gmx output or using GraphPad Prism. Images were created using Pymol for cluster structures.

### MD estimation of NMR chemical shifts

The 15 μs trajectories for WT R1R3_263-311ΔR2_, P270S R1R3_263-311ΔR2_, WT R2R3_295-311_, and P301S R2R3_295-311_ were reduced to contain all protein atoms at a 400 ps timestep for a total of 37,500 time points. The reduced trajectory was used to estimate the alpha-carbon proton chemical shifts using the PPM software with the ensemble parameter and the ANN flag^71,72^. Estimated Hα chemical shifts were then used to predict secondary structure propensities using the neighbor corrected secondary structure propensity calculator^41,42^ (ncSPC) with the same neighbor window of 3 as used for the experimental chemical shifts. Pairwise distance of all protons in the 15 μs ensembles subsampled to a 10ns timestep were calculated using ‘gmx pairdist’ command. Using custom Matlab scripts, these distances were weighted by the dependence of NOE intensities on 1/r^6^, then averaged and the resultant weighted average was transformed into a distance value (μ^−1/6)^ to determine expected NOE distance values that would be representative of our ensemble.

### MD state analysis with Markov State Model (MSM)

MSM of the peptide simulations were built from each peptide 15 μs simulations in ionic solvent using the pyEMMA software. We constructed the MSM of these trajectories to capture the kinetic behavior of the system with the PyEMMA 2.5.12 library^45^ in Python 3.10.9. After the second step of MD simulation WT R1R3_263-311ΔR2_, P270S R1R3_263-311ΔR2_, WT R2R3_295-311_, and P301S R2R3_295-311_, we trimmed the 50 μs trajectory at 100 ps timestep, and each resulting trajectory (5 concatenated trajectories for 4 systems) contains 30001 frames. The MSM featurization step was performed on the basis of the minimum pairwise distance between residues 1-7 (i.e., TENLKHQ vs KDNIKHV) against amyloid motif residues 12-18 (i.e., XVQIVYK) with a total 49 distance pairs with 150001 frames for a single system. We considered the four featurization techniques for model construction: torsion angle, backbone atom positions, backbone atom intra-molecular distances, and minimum pairwise distance between amyloid motifs. The featurization criteria were chosen based on VAMP-2 scores (Variational Approach for Markov Process). We selected the minimum pairwise residue distance over the torsion angles because these motifs covered functional conformational change and reports on interactions across the region of the peptide that differ in sequence. Further dimensionality reduction was performed from a total of 49 pair distance (150001,49) to 2 dimensions (150001,2) using the time-lagged Independent Component Analysis (tICA). TICA identifies the slowest mode of feature space and maximizes the autocorrelation of reduced coordinates for state decomposition. After that, we used the k-means clustering algorithm for the decomposition of reduced conformational space into 200 microstates. Those conformations that belong to the same energy basin in the TICA subspace are clustered into each microstate, and transition occurs quickly among these states. Further, we lumped the microstates into 6 macrostates using PCCA+ to predict the major conformational changes in peptides. The MSM was determined on implied time scale plot, and the lag time 200 (time/step) has been chosen. The Chapman-Kolmogorov test was applied to validate the consistency of probabilities predicted from MSM and MD simulations. The calculated MSM was further coarse-grained into hidden Markov models to calculate the metastable macrostates and transition probabilities. 200 representative structures were computed for each state extracted from the centroid of the most populated microstate. The first 10 of these structures were used as representative models for each state. We calculated the pair distance for specific residue pairs: N- to C-term distance residues 1-18; mid N- to min C-term distance from average of residue pairs 3-17,4-16, and 5-15; C-term distance from residues 7-18; and N-term distance from residues 1-12. Additionally, the turn geometry was estimated from the ratio of the distance of residue 4-12 divided by the distance of residues 7-12. The distance calculation was performed using the ‘gmx pairdist’ between cα atoms of the selected residue from the 200 representative structures of each state, mean distance was reported with +/− SD. Finally, we applied the transition path theory on the macrostates to predict the gross and net flux and major transition pathway between these states. This MSM analysis provides each peptide conformational ensemble's kinetic, thermodynamic, and structural properties. Images were created using PyEMMA graphics output options or Pymol for representative structures.

### Recombinant tauRD and full-length tau production

We utilized several forms of recombinant tau. Recombinant repeat domain tau (tauRD) constructs were purchased from Twist Bioscience encoded into pET-29b(+) vector without tags. Each plasmid was transformed into BL21-Gold (DE3) cells. Cells were grown in 1X Terrific Broth media with 1 μg/ml kanamycin to OD600 of 1.4 and induced with 1 mM isopropyl β-D-1-thiogalactopyranoside (IPTG) for 3 h at 37 °C. The cells were harvested and lysed in 20 mM 2-(N-morpholino) ethane sulfonic acid (MES), 1 mM Ethylenediaminetetraacetic acid (EDTA), 1 mM MgCl, 5 mM β-mercaptoethanol (BME), 1 mM PMSF, pH 6.8, using an Omni Sonic Ruptor 400 with lysate on ice. After sonication, 500 mM NaCl was added to the lysates, which were boiled for 20 minutes before clarifying by centrifugation. The supernatant of the clarified lysate was exchanged into 20 mM MES, 50 mM NaCl, 5 mM BME, and pH 6.8 by dialysis. The dialyzed sample was filtered, applied to a HiTrap SP HP (GE), and eluted with a 50 mM–1 M NaCl gradient. Tau-containing fractions were concentrated on an Amicon-15 concentrator (5KD cutoff) and applied to HiLoad 16/600 Superdex 75 pg (GE) and eluted into 1X PBS (136.5 mM NaCl, 2.7 mM KCl, 10 mM Na_2_HPO_4_, 1.8 mM KH_2_PO_4_, pH 7.4), 10 mM DTT. Aliquots were all stored at −80 °C in 1 X PBS.

The pET-28b-tau plasmid encoding full-length 2N4R WT tau was a gift from Dr. David Eisenberg (UCLA). The plasmid was transformed into BL21-Gold (DE3) cells. Cells were grown in 1X Terrific Broth media to OD600 of 1.4 and induced with 1 mM isopropyl β-D-1-thiogalactopyranoside for 3 h at 37 °C. The cells were harvested and lysed in 50 mM Tris, 500 mM NaCl, 1 mM β-mercaptoethanol, 20 mM imidazole, 1 mM phenylmethylsulfonyl fluoride (PMSF), pH 7.5, using an Omni Sonic Ruptor 400 at 4 °C. The lysates were centrifuged at 13,000 x g for 20 minutes 4 °C, and the supernatant was applied to a Ni-NTA gravity column and eluted with 50 mM Tris, 250 mM NaCl, 1 mM β-mercaptoethanol, and 300 mM imidazole. Eluting fractions containing tau were desalted into 50 mM MES, 50 mM NaCl, and 1 mM β-mercaptoethanol (pH 6.0) by PD-10 column (GE). Exchanged fractions were applied to a HiTrap SP HP (GE) and eluted with a 50 mM–1 M NaCl gradient. Tau-containing fractions were concentrated on an Amicon-15 concentrator (10kDa cutoff) and applied to a HiLoad 16/600 Superdex 200 pg (GE) and eluted into 1X PBS (136.5 mM NaCl, 2.7 mM KCl, 10 mM Na_2_HPO_4_, 1.8 mM KH_2_PO_4_, pH 7.4). Aliquots were all stored at −80 °C in 1X PBS.

### Transmission electron microscopy

An aliquot of 5 μL of the sample was placed onto a glow-discharged Carbon/Formvar-coated 400-mesh copper grid for 1 minute, washed with distilled water, and then negatively stained with 2 μL of 2% uranyl acetate for 1 min. Images were acquired on a Tecnai G^2^ spirit transmission electron microscope (FEI, Hillsboro, OR), serial number D1067, equipped with a LaB_6_ source at 120 kV using a Gatan ultrascan CCD camera.

### Tau aggregation in cells using biosensor cell lines

Stable HEK293T (ATCC CRL-1268) cell line expressing P301S tauRD-Clover and P301S tauRD-Cerulean (from FM5-CMV) were plated at a density of 20,000 cells per well in a 96-well plate 24 h before treatment. At the endpoint of the tauRD ThT fluorescence aggregation assay, after 5 days of incubation with heparin, 25 μL of each sample were pelleted by ultracentrifugation at 100k x g for 1 h. Pellets were resuspended in 25 μL of OptiMEM and diluted to the desired concentration of 1.5 μM, 150 nM, 15 nM. 10 μL of aggregated protein material was mixed with 1 μL lipofectamine and 9 μL Opti-MEM, incubated at RT for 30 min, and added to cell media in technical triplicate for final concentration in each well of 100 nM, 10 nM, 1 nM, and media control.

### Peptide cross-linking mass spectrometry

Preparation of disaggregated peptides was diluted in 1X PBS and cross-linked at a total protein concentration of 75 μM using 15 μg of starting material for WT R1R3_263-311ΔR2_, P270S R1R3_263-311ΔR2_, WT R2R3_295-311_, P301S R2R3_295-311_. The cross-linking reaction was initiated by adding DSS stock solution (200 mM DSS-d0 and -d12, Creative Molecules, dissolved in dimethyl formamide (DMF)) to a final concentration of 1 mM. Samples were incubated at 37 °C for 30 s with 350 RPM shaking. The cross-linking reactions were quenched by adding ammonium bicarbonate to 50 mM final concentration and incubating for 30 minutes at 37 °C while shaking at 350 RPM. Samples were flash-frozen and lyophilized before processing. Samples were processed as 4 technical replicates on a Thermo Orbitrap Fusion Lumos at UTSW Proteomics core. The mass spectrometer was operated in data-dependent mode by selecting the five most abundant precursor ions (m/z 350–1600, charge state 3+ and above) from a preview scan and subjecting them to collision-induced dissociation (normalized collision energy = 35%, 30 ms activation). Fragment ions were detected at low resolution in the linear ion trap. Dynamic exclusion was enabled (repeat count 1, exclusion duration 30 s). Data was processed to identify unmodified, 1 mono-link, 2 mono-links, and loop-links by peak areas. Data was reported as fraction modification normalized to total peak area per replicate.

### Gel-extracted sample preparation for cross-linking mass spectrometry analysis

174 μg of each construct of tauRD (WT, Turn Mutant, Full mutant, P301S, P301S Turn Mutant, and P301S Full Mutant) was diluted in 1X PBS, 10 mM DTT to a final concentration of 58 μM. The cross-linking reaction was conducted by adding disuccinimidyl suberate (DSS, DSS-d0 and -d12, Creative Molecules, dissolved in DMF) to a final concentration of 1 mM for three minutes at 37 °C while shaking at 350 RPM. The reaction was then quenched by adding ammonium bicarbonate to 50 mM final concentration and incubating for 30 minutes at 37 °C while shaking at 350 RPM. Cross-linked samples were resolved on SDS-PAGE gels (NuPAGE^™^, 4 to 12%, Bis-Tris, 1.5 mm), and the monomer band was extracted from the gel for XL-MS analysis using the following protocol. Bands were sliced into 1mm^3^ pieces and washed twice with MQ water, discarding supernatant after each time. Gel pieces were then covered in acetonitrile/50 mM ammonium bicarbonate (mixed in ratio 2:3, v/v) and sonicated for 5 minutes, and the supernatants were discarded each time. This step was repeated twice. Next, gel pieces were washed in 100% acetonitrile until they became white, then the liquid was removed, and gel pieces were dried by lyophilization. Subsequently, gel pieces were covered in 25 mM ammonium bicarbonate with 10 mM DTT and incubated for 1 hour at 56 °C while shaking at 500 RPM. Next, samples were cooled down, supernatants removed, and a freshly prepared solution of 25 mM ammonium bicarbonate with 55 mM iodoacetamide was added, and gel pieces were incubated for 45 minutes in the dark at room temperature. Then, gel pieces were sequentially washed with 25 mM ammonium bicarbonate, 50% acetonitrile, and 100% acetonitrile with quick vertexing after each wash. The liquid was removed, and gel pieces were dried by lyophilization. Trypsin digestion was initiated by adding 1:10 (trypsin to protein, m/m) mass spectrometry grade trypsin (New England Biolabs #P8101S) in 50 mM ammonium bicarbonate, and then the reaction was incubated overnight at 37 °C while shaking at 500 RPM. Digestion was stopped by adding 2% (v/v) formic acid, and supernatants were collected. Peptides were additionally extracted by covering gel pieces in MQ water/acetonitrile/formic acid (50:50:0.1, v/v/v), incubated for 15 minutes at 37 °C while shaking at 500 RPM, and then sonicated for 5 minutes. This step was repeated twice, and collected supernatants were pooled, snap-frozen, and lyophilized. Peptides were resuspended in 50 mM ammonium bicarbonate/acetonitrile/formic acid (93:5:2), further purified by solid phase extraction using Sep-Pak tC18 cartridges (Waters #WAT054960), snap frozen and lyophilized. As described above for peptide cross-linking mass spectrometry, samples were resuspended and analyzed at the UTSW Proteomics core.

### Analysis of mass spectrometry data

Thermo.raw files were converted to the open.mzXML format using msconvert (proteowizard.sourceforge.net) and analyzed using an in-house version of xQuest^53^. Spectral pairs with a precursor mass difference of 12.075321 Da were extracted and searched against the respective FASTA databases containing tau (TAU_HUMAN P10636-8) or designed constructs with and without P301S mutations. xQuest settings were as follows: Maximum number of missed cleavages (excluding the cross-linking site) = 2, peptide length = 5–50 aa, fixed modifications = carbamidomethyl-Cys (mass shift = 57.021460 Da), variable modification = oxidation of methionine (mass shift = 15.99491 Da), mass shift of the light crosslinker = 138.0680796 Da, mass shift of mono-links = 156.0786442 and 155.0964278 Da, MS1 tolerance = 10 ppm, MS2 tolerance = 0.2 Da for common ions and 0.3 Da for cross-link ions, search in ion-tag mode. Post-search manual validation and filtering were performed using the following criteria: xQuest score >25, FDR <0.05, mass error between − 2.2 and + 3.8 ppm, %TIC >10, and a minimum peptide length of six aa. FDRs for the identified cross-links were estimated using xProphet^73^. The five replicate data sets were compared, and cross-links in five out of five data sets were used to generate a consensus data set. Normalized mono-links and cross-link data with information on cross-linked residue positions and nseen (frequency) were visualized using customized Matlab scripts. Hierarchical clustering was done using Matlab tools clustergram and linkage to build a linkage dendrogram trees across identified loop-links/cross-links for all 6 constructs.

### Tau aggregation in cells using mEOS biosensor cell lines

FM5-CMV constructs of P301S, P301S_V300Q, P301S_S305K, P301S Turn mutant, P301S Full mutant tauRD fused to mEOS3.2 at the C-terminal were used for cell expression. The virus of each construct was produced in Lenti-X^™^ 293 T Cell Line (Takara, Cat. #632180) and previously described^74^. Specifically, 300 ng PSP helper plasmid, 100 ng VSVG, and 100 ng of the plasmid of interest were transiently co-transfected using 1.875 μL TransIT (Mirus Bio), and Opti-MEM (Gibco) were mixed to a final volume of ~30 μL. Media (10% FBS, 1% Pen/Strep, 1% GlutaMax in Dulbecco’s modified Eagle’s medium) containing the virus was collected after 48 h and centrifuged at 1000 RPM for 5 min to remove debris and dead cells. For transduction, 10-50 μL of virus suspension was added to HEK293T (ATCC CRL-1268), and cells were grown in virus-containing media for 72 h before expanding to the most confluent condition. Cells were harvested from a 10 cm dish with 0.05% trypsin, resuspended in flow cytometry buffer (HBSS plus 1% FBS and 1 mM EDTA), and subjected to FACS (Sony Biotechnology). Populations with FITC-A signal above 10^4^ were collected for high-expressing cells. Following FACS and expansion, cells were maintained as a polyclonal line. All stable cell lines were amplified, frozen, and stored in liquid nitrogen until use.

For seeding experiments, cells were plated in 96-well plates at 20,000 cells/well in 130 μL of media. 24 h later, the cells were treated with 20 μL of fibril treatment with a dilution series in technical triplicates. Briefly, treatments were composed of heparin-induced 2N4R recombinant tau fibrils (8 μM monomer equivalent), sonicated for 5 minutes at an amplitude of 65 and 30 s off/on a Q700 Sonicator (QSonica). Sonicated fibrils were diluted to the desired concentration at 10 μL, mixed with 1 μL lipofectamine and 9 μL Opti-MEM, incubated at RT for 30 min per condition, and replicated. After incubation, samples were added to cell media for final concentration in the well of 300 nM, 100 nM, 30 nM, 10 nM, 3 nM, 1 nM, and media control. After 48 hours, cells were harvested with 0.05% trypsin and then fixed in 2% paraformaldehyde (Electron Microscopy Services) for 10 min at room temperature, after which PFA was removed, and cells were resuspended in 1X PBS. For the initial experiment, the optimal time of photoconversion was determined by exposing cells to UV for 0-60 minutes and measuring the mEOS red-to-green ratio, which determined 20 minutes as the optimal Red/Green ratio used for all subsequent experiments.

### Flow cytometry

A BD LSRFortessa was used to perform FRET flow cytometry, mCerulean3 and FRET signal was measured by exciting with the 405 nm laser, and fluorescence was captured with a 405/50 nm and 525/50 nm filter, respectively. mClover3 signal was measured by exciting cells with a 488 nm laser, and fluorescence was captured with a 525/50 nm filter. To quantify FRET, we used a gating strategy where mCerulean3 bleed through into the mClover3, and FRET channels were compensated using FlowJo analysis software, as described previously^75^. Subsequently, we created a final bivariate plot of FRET vs. Cerulean and introduced a triangular gate to assess the number of FRET-positive cells. This FRET gate was adjusted to biosensor cells that received lipofectamine alone and are thus FRET-negative. FRET signal is defined as the percentage of FRET-positive cells in all analyses. For each experiment, 10,000 mClover3/mCerulean3 double-positive cells per replicate were analyzed, and each condition was analyzed in triplicate. Data analysis was performed using FlowJo v10 software (Treestar).

For the mEOS3.2 cell expression system, tauRD-mEOS biosensor cells were first photoconverted under UV for 20 min. 10,000 singlet events corresponding to the donor (non-photoconverted mEOS3.2) and acceptor (photoconverted mEOS3.2) positive cells were collected for each sample. Non-photoconverted mEOS3.2 was collected at Alexa Flour 488 channel. FCS files were exported from the BD FACSDiva data collection software and analyzed using FlowJo v10 software (Treestar). Compensation was manually applied to correct donor bleed-through into the FRET channel guided by a sample with non-aggregated and photoconverted tauRD-mEOS. Samples were gated on the acceptor intensity such that cells with similar concentrations of tauRD-mEOS were analyzed to mitigate the contribution of differences in concentration leading to apparent changes in the fraction of FRET-positive cells in each condition. FRET-positive cells were quantified by gating double-positive singlet events with a ratio of FRET to donor signal higher than that of a population of tauRD-mEOS photoconverted cells without aggregates.

### Microtubule Polymerization assay

Experiments were carried out following the Cytoskeleton Tubulin Polymerization Fluorescence-based Assay (BK011P) protocol. TauRD constructs in 20 mM MES and 10 mM DTT buffer were mixed with 99.9% purified tubulin at 2 mg/ml in 80 mM PIPES, 2 mM MgCl, 0.5 mM EGTA, pH 6.9, 10 μM fluorescent reporter to a final concentration at 10 μM. 3 μM paclitaxel solution was used as a positive control. All conditions were done in triplicates at 37 °C. ThT kinetic scans were run every minute on a Tecan Spark plate reader at 350 nm Ex (10 nm bandwidth) and 435 nm Em (20 nm bandwidth). Blank wells containing buffer and tubulin were subtracted from experimental values. The data were min-max normalized to the sample with maximal signal (taxol positive control), and the data was used for non-linear regression to determine the intensity max and the t_1/2_max for assessing polymerization time. Column effects using 2way ANOVA were calculated for the intensity max and t_1/2_max using GraphPad Prism v8.0-10.0.

### MT:tau structure Analysis

The synthetic tau (R2X4) model bound to microtubules (MT) from PDB ID 6CVN was rebuilt to have the 4R tauRD designed sequence using the Rosetta threading protocol with bilateral loop building to include the missing residue from the structures^22^. For each sequence, 2,500 models of only tau without the MT were produced, constrained by the initial template of the tau structures. Models were then realigned with the α/β/α tubulin, and the tau chain was trimmed to contain only tau repeats 2 and 3 (275-331) with backbone atoms only (atoms NH, N, CA, CB, and C─O). Each structure was minimized using the Rosetta protocol with the lbfgs_armijo_nonmonotone algorithm and a minimum tolerance of 0.001 (https://www.rosettacommons.org/software). The resulting minimized structures were scored as a complex using the InterfaceAnalyzer protocol, pre-packing separate interfaces and tubulin chains comprising interface 1 and tau as interface 2. The resulting structures were compared to the lowest scoring structure to determine the built model RMSD using the Rosetta scoring function. All model building and analysis were performed on UTSW’s BioHPC computing cluster. All plots were generated with GraphPad Prism. Images were created using Pymol.

### Statistics and Reproducibility

All statistics were calculated using GraphPad Prism v8.0-10.0. Three independent ThT experiments were run for each condition. The data were normalized to the highest amplitude, and averages and standard deviations were plotted. Plots were fitted to a non-linear regression model, from which t_1/2_ values were derived. t_1/2_ error represents a 95% CI. TEM grids of endpoint ThT samples were screeded and imaged a minimum of 4 times to obtain representative images. Flow cytometry cell aggregation was conducted in three independent experiments, whose values are plotted. Error bars represent 95% CI.

## Data Availability

All MD, NMR, ThT, XL-MS, MT stabilization, MT:tau modeling, and cell-based aggregation data are available as source data 1, source data 2, source data 3, source data 4, source data 5, source data 6, and source data 7, respectively. PDB IDs used in the study include 2MZ7 [https://doi.org/10.2210/pdb2MZ7/pdb], 6CVN [https://doi.org/10.2210/pdb6CVN/pdb] and 8Q92 [https://doi.org/10.2210/pdb8q92/pdb].

## Code availability

All MD simulations were performed using Gromacs v5.0.4 (available at http://www.gromacs.org). Markov State Model analysis was performed using pyEMMA v2.5.7 (http://emma-project.org). NMR spectra were processed, assigned, and visualized using NMRpipe (https://www.ibbr.umd.edu/nmrpipe/install.html), Cara v1.9.1 (http://cara.nmr.ch/doku.php) and Sparky 3 (https://www.cgl.ucsf.edu/home/sparky/). XL-MS data was analyzed using xQuest v2.1.5 (https://gitlab.ethz.ch/leitner_lab/xquest_xprophet). All MT:tau simulations were performed using Rosetta v3.12 (available at https://www.rosettacommons.org).

## References

[1] Vaquer-Alicea J., Diamond M. I. & Joachimiak L. A. Tau strains shape disease. Acta Neuropathol 142, 57–71 (2021). 10.1007/s00401-021-02301-7 33830330 PMC8217038

[2] Gao Y.-L. Tau in neurodegenerative disease. Annals of Translational Medicine 6 (2018/05). 10.21037/atm.2018.04.23 PMC5994507 29951497

[3] Orr M. E., Sullivan A. C. & Frost B. A Brief Overview of Tauopathy: Causes, Consequences, and Therapeutic Strategies. Trends in Pharmacological Sciences 38, 637–648 (2017). 10.1016/j.tips.2017.03.011 28455089 PMC5476494

[4] Fitzpatrick A. W. P. Cryo-EM structures of tau filaments from Alzheimer's disease. Nature 547, 185–190 (2017). 10.1038/nature23002 28678775 PMC5552202

[5] Falcon B. Novel tau filament fold in chronic traumatic encephalopathy encloses hydrophobic molecules. Nature 568, 420–423 (2019). 10.1038/s41586-019-1026-5 30894745 PMC6472968

[6] Lövestam S K. F., van Knippenberg B, Kotecha A, Murzin AG, Goedert M, Scheres SHW. . Assembly of recombinant tau into filaments identical to those of Alzheimer's disease and chronic traumatic encephalopathy. eLife 11 (2022). 10.7554/eLife.76494 PMC8983045 35244536

[7] Han Z. Z., Kang S. G., Arce L. & Westaway D. Prion-like strain effects in tauopathies. Cell Tissue Res 392, 179–199 (2023). 10.1007/s00441-022-03620-1 35460367 PMC9034081

[8] Kaufman S. K., Thomas T. L., Del Tredici K., Braak H. & Diamond M. I. Characterization of tau prion seeding activity and strains from formaldehyde-fixed tissue. Acta Neuropathol Commun 5, 41 (2017). 10.1186/s40478-017-0442-8 28587664 PMC5461712

[9] Mirbaha H. Inert and seed-competent tau monomers suggest structural origins of aggregation. Elife 7 (2018). 10.7554/eLife.36584 PMC6039173 29988016

[10] Sharma A. M., Thomas T. L., Woodard D. R., Kashmer O. M. & Diamond M. I. Tau monomer encodes strains. eLife 7 (2018). 10.7554/elife.37813 PMC6289568 30526844

[11] Mirbaha H. Seed-competent tau monomer initiates pathology in a tauopathy mouse model. J Biol Chem 298, 102163 (2022). 10.1016/j.jbc.2022.102163 35750209 PMC9307951

[12] Hou Z., Chen D., Ryder B. D. & Joachimiak L. A. Biophysical properties of a tau seed. Sci Rep 11, 13602 (2021). 10.1038/s41598-021-93093-z 34193922 PMC8245522

[13] Chen D. Tau local structure shields an amyloid-forming motif and controls aggregation propensity. Nat Commun 10, 2493 (2019). 10.1038/s41467-019-10355-1 31175300 PMC6555816

[14] Mullapudi V. Network of hotspot interactions cluster tau amyloid folds. Nat Commun 14, 895 (2023). 10.1038/s41467-023-36572-3 36797278 PMC9935906

[15] van der Kant R., Louros N., Schymkowitz J. & Rousseau F. Thermodynamic analysis of amyloid fibril structures reveals a common framework for stability in amyloid polymorphs. Structure 30, 1178–1189 e1173 (2022). 10.1016/j.str.2022.05.002 35609599

[16] Lövestam S., Li D., Wagstaff J. L., Kotecha A., Kimanius D., McLaughlin S. H., Murzin A. G., Freund S. M., Goedert M., & Scheres S. H. Disease-specific tau filaments assemble via polymorphic intermediates. Nature (2023). 10.1038/s41586-023-06788-w PMC10764278 38030728

[17] Chakraborty P. Acetylation discriminates disease-specific tau deposition. Nat Commun 14, 5919 (2023). 10.1038/s41467-023-41672-1 37739953 PMC10517010

[18] Li L N. B., Mullapudi V, Li Y, Saelices L, Joachimiak LA. . Disease-associated patterns of acetylation stabilize tau fibril formation. Structure (London, England : 1993) 31 (2023). 10.1016/j.str.2023.05.020 PMC10527703 37348495

[19] Arakhamia T. Posttranslational Modifications Mediate the Structural Diversity of Tauopathy Strains. Cell 180, 633–644 e612 (2020). 10.1016/j.cell.2020.01.027 32032505 PMC7491959

[20] Wesseling H. Tau PTM Profiles Identify Patient Heterogeneity and Stages of Alzheimer's Disease. Cell 183, 1699–1713 e1613 (2020). 10.1016/j.cell.2020.10.029 33188775 PMC8168922

[21] Lee G., Neve R. L. & Kosik K. S. The microtubule binding domain of tau protein. Neuron 2, 1615–1624 (1989). 10.1016/0896-6273(89)90050-0 2516729

[22] Kellogg E. H. Near-atomic model of microtubule-tau interactions. Science 360, 1242–1246 (2018). 10.1126/science.aat1780 29748322 PMC6225777

[23] Barré P E. D. Structural transitions in tau k18 on micelle binding suggest a hierarchy in the efficacy of individual microtubule-binding repeats in filament nucleation. Protein science : a publication of the Protein Society 22 (2013). 10.1002/pro.2290 PMC3832040 23740819

[24] Lathuilière A. Motifs in the tau protein that control binding to microtubules and aggregation determine pathological effects. Scientific Reports 7 (2017). 10.1038/s41598-017-13786-2 PMC5648870 29051562

[25] Goedert M. & Spillantini M. G. Tau mutations in frontotemporal dementia FTDP-17 and their relevance for Alzheimer’s disease. Biochimica et Biophysica Acta (BBA) - Molecular Basis of Disease 1502, 110–121 (2000). 10.1016/s0925-4439(00)00037-5 10899436

[26] van Swieten J. & Spillantini M. G. Hereditary frontotemporal dementia caused by Tau gene mutations. Brain Pathol 17, 63–73 (2007). 10.1111/j.1750-3639.2007.00052.x 17493040 PMC8095608

[27] Kinoshita J. & Clark T. Alzforum. Methods Mol Biol 401, 365–381 (2007). 10.1007/978-1-59745-520-6_19 18368375

[28] Bugiani O. Frontotemporal dementia and corticobasal degeneration in a family with a P301S mutation in tau. J Neuropathol Exp Neurol 58, 667–677 (1999). 10.1097/00005072-199906000-00011 10374757

[29] Liu F. & Gong C.-X. Tau exon 10 alternative splicing and tauopathies. Molecular Neurodegeneration 3 (2008). 10.1186/1750-1326-3-8 PMC2483273 18616804

[30] Chen S., Townsend K., Goldberg T. E., Davies P. & Conejero-Goldberg C. MAPT Isoforms: Differential Transcriptional Profiles Related to 3R and 4R Splice Variants. Journal of Alzheimer's Disease 22 (2010). 10.3233/JAD-2010-101155 PMC3322427 20930284

[31] Schoch K. M. Increased 4R-Tau Induces Pathological Changes in a Human-Tau Mouse Model. Neuron 90, 941–947 (2016). 10.1016/j.neuron.2016.04.042 27210553 PMC5040069

[32] Limorenko G. & Lashuel H. A. To target Tau pathologies, we must embrace and reconstruct their complexities. Neurobiol Dis 161, 105536 (2021). 10.1016/j.nbd.2021.105536 34718129

[33] Mummery C. J. Tau-targeting antisense oligonucleotide MAPT(Rx) in mild Alzheimer's disease: a phase 1b, randomized, placebo-controlled trial. Nat Med 29, 1437–1447 (2023). 10.1038/s41591-023-02326-3 37095250 PMC10287562

[34] Yoshiyama Y. Synapse Loss and Microglial Activation Precede Tangles in a P301S Tauopathy Mouse Model. Neuron 53 (2007/02/01). 10.1016/j.neuron.2007.01.010 17270732

[35] Holmes B. B. Proteopathic tau seeding predicts tauopathy in vivo. Proc Natl Acad Sci U S A 111, E4376–4385 (2014). 10.1073/pnas.1411649111 25261551 PMC4205609

[36] Macdonald J. A. Assembly of transgenic human P301S Tau is necessary for neurodegeneration in murine spinal cord. Acta Neuropathologica Communications 2019 7:17 (2019-03-18). 10.1186/s40478-019-0695-5 PMC6421678 30885267

[37] von Bergen M. Mutations of tau protein in frontotemporal dementia promote aggregation of paired helical filaments by enhancing local beta-structure. J Biol Chem 276, 48165–48174 (2001). 10.1074/jbc.M105196200 11606569

[38] Sahayaraj A. E., Viswanathan R., Pinhero F., Abdul Vahid A. & Vijayan V. Sequence-Dependent Conformational Properties of PGGG Motif in Tau Repeats: Insights from Molecular Dynamics Simulations of Narrow Pick Filament. ACS Chem Neurosci 14, 136–147 (2023). 10.1021/acschemneuro.2c00602 36512636

[39] Schweighauser M. Cryo-EM structures of tau filaments from the brains of mice transgenic for human mutant P301S Tau. Acta Neuropathologica Communications 2023 11:1 11 (2023-10-05). 10.1186/s40478-023-01658-y PMC10552433 37798679

[40] Stelzl L. S. Global Structure of the Intrinsically Disordered Protein Tau Emerges from Its Local Structure. JACS Au 2, 673–686 (2022). 10.1021/jacsau.1c00536 35373198 PMC8970000

[41] Tamiola K. & Mulder F. A. Using NMR chemical shifts to calculate the propensity for structural order and disorder in proteins. Biochem Soc Trans 40, 1014–1020 (2012). 10.1042/BST20120171 22988857

[42] Tamiola K., Acar B. & Mulder F. A. A. Sequence-Specific Random Coil Chemical Shifts of Intrinsically Disordered Proteins. Journal of the American Chemical Society 132 (December 3, 2010). 10.1021/ja105656t 21128621

[43] Kawasaki R T. S. Impact of the Hereditary P301L Mutation on the Correlated Conformational Dynamics of Human Tau Protein Revealed by the Paramagnetic Relaxation Enhancement NMR Experiments. International journal of molecular sciences 21 (2020). 10.3390/ijms21113920 PMC7313075 32486218

[44] Lindorff-Larsen K. Improved side-chain torsion potentials for the Amber ff99SB protein force field. Proteins 78, 1950–1958 (2010). 10.1002/prot.22711 20408171 PMC2970904

[45] Scherer M. K. PyEMMA 2: A Software Package for Estimation, Validation, and Analysis of Markov Models. J Chem Theory Comput 11, 5525–5542 (2015). 10.1021/acs.jctc.5b00743 26574340

[46] Holmes B. B. & Diamond M. I. Prion-like properties of Tau protein: the importance of extracellular Tau as a therapeutic target. J Biol Chem 289, 19855–19861 (2014). 10.1074/jbc.R114.549295 24860099 PMC4106306

[47] Leitner A. The molecular architecture of the eukaryotic chaperonin TRiC/CCT. Structure 20, 814–825 (2012). 10.1016/j.str.2012.03.007 22503819 PMC3350567

[48] Leitner A. Chemical cross-linking/mass spectrometry targeting acidic residues in proteins and protein complexes. Proc Natl Acad Sci U S A 111, 9455–9460 (2014). 10.1073/pnas.1320298111 24938783 PMC4084482

[49] Lasker K. Molecular architecture of the 26S proteasome holocomplex determined by an integrative approach. Proc Natl Acad Sci U S A 109, 1380–1387 (2012). 10.1073/pnas.1120559109 22307589 PMC3277140

[50] Chlebowicz J. Saturation mutagenesis of alpha-synuclein reveals monomer fold that modulates aggregation. Sci Adv 9, eadh3457 (2023). 10.1126/sciadv.adh3457 37889966 PMC10610913

[51] Hou Z. DnaJC7 binds natively folded structural elements in tau to inhibit amyloid formation. Nat Commun 12, 5338 (2021). 10.1038/s41467-021-25635-y 34504072 PMC8429438

[52] Wydorski P. M. Dual domain recognition determines SARS-CoV-2 PLpro selectivity for human ISG15 and K48-linked di-ubiquitin. Nat Commun 14, 2366 (2023). 10.1038/s41467-023-38031-5 37185902 PMC10126577

[53] Rinner O. Identification of cross-linked peptides from large sequence databases. Nature Methods 2008 5:4 5 (2008-03-09). 10.1038/nmeth.1192 PMC2719781 18327264

[54] Xia Y. Impaired tau–microtubule interactions are prevalent among pathogenic tau variants arising from missense mutations. The Journal of Biological Chemistry 294 (2019). 10.1074/jbc.RA119.010178 PMC6885647 31653695

[55] Kadavath H. Tau stabilizes microtubules by binding at the interface between tubulin heterodimers. Proc Natl Acad Sci U S A 112, 7501–7506 (2015). 10.1073/pnas.1504081112 26034266 PMC4475932

[56] Kadavath H. Folding of the Tau Protein on Microtubules. Angew Chem Int Ed Engl 54, 10347–10351 (2015). 10.1002/anie.201501714 26094605

[57] Sündermann F., Fernandez M.-P. & Morgan R. O. An evolutionary roadmap to the microtubule-associated protein MAP Tau. BMC Genomics 17 (2016). 10.1186/s12864-016-2590-9 PMC4815063 27030133

[58] El Mammeri N., Dregni A. J., Duan P., Wang H. K. & Hong M. Microtubule-binding core of the tau protein. Sci Adv 8, eabo4459 (2022). 10.1126/sciadv.abo4459 35857846 PMC9299549

[59] Strang K. H. Distinct differences in prion-like seeding and aggregation between Tau protein variants provide mechanistic insights into tauopathies. J Biol Chem 293, 4579 (2018). 10.1074/jbc.AAC118.002657 29572329 PMC5868274

[60] Chen D. FTD-tau S320F mutation stabilizes local structure and allosterically promotes amyloid motif-dependent aggregation. Nat Commun 14, 1625 (2023). 10.1038/s41467-023-37274-6 36959205 PMC10036635

[61] Gerasimavicius L., Livesey B. J. & Marsh J. A. Loss-of-function, gain-of-function and dominant-negative mutations have profoundly different effects on protein structure. Nature Communications 13 (2022). 10.1038/s41467-022-31686-6 PMC9259657 35794153

[62] Pounot K., Piersson C., Goring A., Weik M., Han S., Fichou Y. Mutations in tau protein promote aggregation by favoring extended conformations. (2023). https://doi.org/10.1101/2023.05.12.540512 PMC10806773 38274251

[63] Bali S. J., Modifying L. Amyloid Motif Aggregation Through Local Structure - PubMed. Methods in molecular biology (Clifton, N.J.) 2340 (2022). 10.1007/978-1-0716-1546-1_15 35167081

[64] Hinds M. G. & Norton R. S. NMR Spectroscopy of Peptides and Proteins. (1994). 10.1385/0-89603-274-4:131 7697108

[65] Delaglio F G. S., Vuister GW, Zhu G, Pfeifer J, Bax A. NMRPipe: a multidimensional spectral processing system based on UNIX pipes. Journal of biomolecular NMR 6 (1995). 10.1007/BF00197809 8520220

[66] Lee W T. M., Markley JL, NMRFAM-SPARKY: enhanced software for biomolecular NMR spectroscopy. Bioinformatics (Oxford, England) 31 (2015). 10.1093/bioinformatics/btu830 PMC4393527 25505092

[67] Berendsen H. J. C., Postma J. P. M., Van Gunsteren W. F., Dinola A. & Haak J. R. Molecular dynamics with coupling to an external bath. The Journal of Chemical Physics 81, 3684–3690 (1984). 10.1063/1.448118

[68] Parrinello M. & Rahman A. Polymorphic transitions in single crystals: A new molecular dynamics method. Journal of Applied Physics 52, 7182–7190 (1981). 10.1063/1.328693

[69] Feenstra K. A. & Hess B. B., H. J. C. . Improving efficiency of large time-scale molecular dynamics simulations of hydrogen-rich systems. Journal of Computational Chemistry (1999). 10.1002/(SICI)1096-987X(199906)20:8<786::AID-JCC5>3.0.CO;2-B 35619462

[70] Darden T., York D. & Pedersen L. Particle mesh Ewald: An N·log(N) method for Ewald sums in large systems. The Journal of Chemical Physics 98 (1993/06/15). 10.1063/1.464397

[71] Li DW B. R. PPM: a side-chain and backbone chemical shift predictor for the assessment of protein conformational ensembles. Journal of biomolecular NMR 54 (2012). 10.1007/s10858-012-9668-8 22972619

[72] Li D B. R. PPM_One: a static protein structure based chemical shift predictor. Journal of biomolecular NMR 62 (2015). 10.1007/s10858-015-9958-z 26091586

[73] Walzthoeni T. False discovery rate estimation for cross-linked peptides identified by mass spectrometry. Nat Methods 9, 901–903 (2012). 10.1038/nmeth.2103 22772729

[74] Stopschinski B. E. Specific glycosaminoglycan chain length and sulfation patterns are required for cell uptake of tau versus alpha-synuclein and beta-amyloid aggregates. J Biol Chem 293, 10826–10840 (2018). 10.1074/jbc.RA117.000378 29752409 PMC6036193

[75] Furman J. L., Holmes B. B. & Diamond M. I. Sensitive Detection of Proteopathic Seeding Activity with FRET Flow Cytometry. J Vis Exp, e53205 (2015). 10.3791/53205 PMC4692784 26710240

